# Therapeutic Emergence of Rhein as a Potential Anticancer Drug: A Review of Its Molecular Targets and Anticancer Properties

**DOI:** 10.3390/molecules25102278

**Published:** 2020-05-12

**Authors:** Sahu Henamayee, Kishore Banik, Bethsebie Lalduhsaki Sailo, Bano Shabnam, Choudhary Harsha, Satti Srilakshmi, Naidu VGM, Seung Ho Baek, Kwang Seok Ahn, Ajaikumar B Kunnumakkara

**Affiliations:** 1 Cancer Biology Laboratory and DBT-AIST International Laboratory for Advanced Biomedicine (DAILAB), Department of Biosciences and Bioengineering, Indian Institute of Technology (IIT) Guwahati, Assam 781039, India; henamayeesahu@yahoo.com (S.H.); kishore.banik@iitg.ac.in (K.B.); b.sailo@iitg.ac.in (B.L.S.); bano176106104@iitg.ac.in (B.S.); harsha.choudhary@iitg.ac.in (C.H.); 2 Department of Pharmacology & Toxicology, National Institute of Pharmaceutical Education and Research (NIPER, Guwahati), Assam 781125, India; srilakshmisatthi@gmail.com (S.S.); vgmnaidu@gmail.com (N.V.); 3 College of Korean Medicine, Dongguk University, 32 Dongguk-ro, Ilsandong-gu, Goyang-si, Gyeonggi-do 10326, Korea; baekone99@gmail.com; 4 Department of Science in Korean Medicine, Kyung Hee University, 24 Kyungheedae-ro, Dongdaemun-gu, Seoul 02447, Korea

**Keywords:** rhein, cancer, phytochemical, molecular targets, chemoprevention, chemotherapy

## Abstract

According to the World Health Organization (WHO), cancer is the second-highest cause of mortality in the world, and it kills nearly 9.6 million people annually. Besides the fatality of the disease, poor prognosis, cost of conventional therapies, and associated side-effects add more burden to patients, post-diagnosis. Therefore, the search for alternatives for the treatment of cancer that are safe, multi-targeted, effective, and cost-effective has compelled us to go back to ancient systems of medicine. Natural herbs and plant formulations are laden with a variety of phytochemicals. One such compound is rhein, which is an anthraquinone derived from the roots of *Rheum* spp. and *Polygonum multiflorum*. In ethnomedicine, these plants are used for the treatment of inflammation, osteoarthritis, diabetes, and bacterial and helminthic infections. Increasing evidence suggests that this compound can suppress breast cancer, cervical cancer, colon cancer, lung cancer, ovarian cancer, etc. in both in vitro and in vivo settings. Recent studies have reported that this compound modulates different signaling cascades in cancer cells and can prevent angiogenesis and progression of different types of cancers. The present review highlights the cancer-preventing and therapeutic properties of rhein based on the available literature, which will help to extend further research to establish the chemoprotective and therapeutic roles of rhein compared to other conventional drugs. Future pharmacokinetic and toxicological studies could support this compound as an effective anticancer agent.

## 1. Introduction

Cancer is the second leading cause of death globally, killing around 9.6 million people annually [1]. Despite the significant advances in the field of cancer therapy, major limitations such as drug inefficacy, drug resistance, distant metastasis, associated side-effects, and toxicity hinder the use of chemotherapeutic approaches [2,3,4,5,6,7,8,9]. Therefore, there is an urgent need to discover novel therapeutic agents, as the number of cancer-related deaths will increase dramatically in the coming years [1]. It is now evident that naturally derived bioactives have been a boon to human civilization since time immemorial. The Ayurveda, the Chinese Pharmacopoeia (2005), Unani and other Indian systems of medicine demonstrate the importance of these multi-targeted phytochemicals for the prevention and treatment of different diseases in humans [10,11]. Besides, there is also evidence that our daily diet has a significant protective role, guarding us against oxidative stress and various disorders [12,13,14,15,16,17,18,19,20,21,22]. It is well established that natural compounds rich in antioxidants can activate different survival pathways and protect normal cells from the adverse effects of anticancer therapies [23,24]. Additionally, many of these dietary ingredients are known to directly or indirectly increase the actions of many chemotherapeutic drugs, which in turn enhances their therapeutic potential, as evident from many preclinical and clinical studies [25,26,27,28,29,30,31,32]. Again, the chemoprotective and chemosensitizing effects of natural products have widened the research arena to include compounds beyond conventional anticancer drugs [33,34,35,36,37,38]. One such compound is rhein, a naturally derived aglycone from rhubarb leaves. Traditional practices show that the rhubarb plant (*Rheum officinale*) was widely prescribed due to its anticathartic and antistomachic properties. Its therapeutic potential also includes antibacterial, antidiabetic, antiinflammatory and anticancer activities [39,40,41,42,43,44]. Additionally, one of the metabolic precursors of rhein, diacerein, has shown significant results in easing pain and improving matrix synthesis in the treatment of osteoarthritis [45]. A large number of preclinical studies have demonstrated the anticancer activities of rhein against breast cancer, cervical cancer, nasopharyngeal cancer, tongue cancer, pancreatic cancer, ovarian cancer, and hepatocellular carcinoma, in addition to its pro-apoptotic, antiproliferative, and antiangiogenic properties [46,47,48,49,50,51,52,53,54,55,56,57]. In addition, preclinical studies have shown that as an antineoplastic compound, rhein potentiated the cytotoxic effect of chemotherapeutic drugs, minimized side-effects, enhanced tolerability, and reduced multidrug resistance [57]. Although this anthraquinone glycoside has a broad spectrum of therapeutic potential, it shows poor systemic ability and remains unexplored due to its highly hydrophobic structure [58]. However, there are formulations that can overcome these issues [59,60,61]. The broad aim of this article was to provide an overall framework describing the nature, chemistry, ethnopharmacological uses, biological activities, molecular targets, and the chemoprotective, chemopreventive and therapeutic potential of rhein in different cancers.

## 2. Rhein in Nature

**Figure 1:**
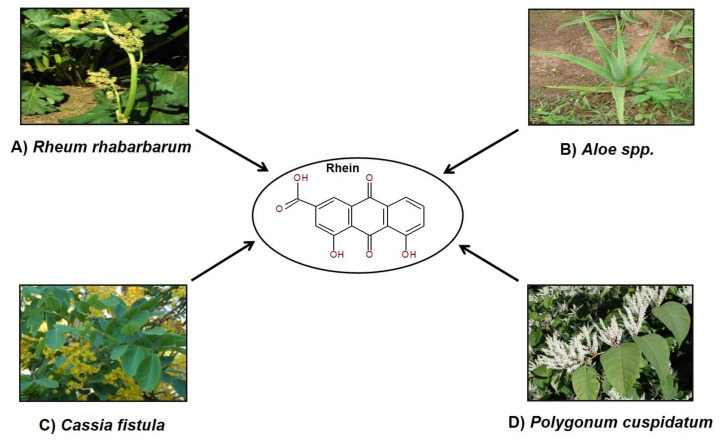
Sources of rhein: (**A**) *Rheum rhabarbarum* (Kay Yatskievych/ www.discoverlife.org); (**B**) *Aloe spp.* (Pankaj Oudhia/ www.discoverlife.org); (**C**) *Cassia fistula* (John Pickering/ www.discoverlife.org); (**D**) *Polygonum cuspidatum* (Les Mehrhoff/ www.discoverlife.org).

Nature offers many safe, effective, and affordable curatives for many chronic ailments, which are mainly obtained from plants. These phytochemicals are extracted from different parts of plants and many act as dietary supplements and have significant health benefits [62,63,64]. The compound rhein which has shown many medicinal properties, is one of the major phytochemical components of plants like *Aloe barbadensis* (Family Asphodelaceae), *Rheum* spp., *Polygonum multiflorum*, *P. cuspidatum* (Family Polygonaceae), *Cassia occidentalis* (Family Fabaceae), etc., as described later [65,66]. Various sources of rhein are depicted in Figure 1. Studies have shown that the Chinese medicine Da Huang or Chinese rhubarb, used for treating inflammation in humans, is composed of three different *Rheum* species, i.e., *R. palmatum* L., *R. tanguticum*, and *R. officinale*, all of which contain rhein [67].

The foliar parts of cultivated species of *Rheum* are usually edible [68]. Potential larvicidal and anticancerous activities were observed in isolates of rhein from extracts of yellow *Cassia fistula* flowers [69,70].

## 3. Ethnopharmacological Uses of Plants Containing Rhein

Plants containing rhein have been used in different systems of traditional medicine for the prevention and treatment of different diseases. The 1000-year-old Chinese Pharmacopoeia suggests the use of the Rhei Rhizoma or rhubarb components as a laxative because they stimulate the secretion of bile into the intestines and support the expulsion of toxic waste matter. In addition, the components of rhubarb are also involved in hepatoprotection and antibacterial activity [71,72]. The Chinese Pharmacopoeia (1997) suggests a potential use of the herb *P. multiflorum* for blood cleansing and improving blood circulation [73]. The root extracts of this plant, popularly known as Heshouwu in China, are used for their antiobesity properties [74]. The use of the herb *P. multiflorum* was cited in the literature of the Tang dynasty and the Song dynasty and the 2010 edition of the Chinese Pharmacopoeia. Its formulation with other herbs reduces its toxicity and acts over several human health disorders. There have been 242 patented formulations of the plant *P. multiflorum* with other herbs such as *Radix rehmanniae*, *R. astragalus*, *R. ophiopogonis*, *Salviae miltiorrhizae*, and *Angelica sinensis*, all with different pharmacological properties. The associated studies demonstrated that combining *P. multiflorum* with other herbs enhanced its beneficial effects on human health, such as longer serum retention of its bioactive compounds and decreased toxicity [75]. In a clinical study on 312 patients, the alcoholic extracts of rhubarb showed a significant effect in the treatment of gastric and duodenal ulcer bleeding [76]. Another traditional Chinese medicine, San-Huang-Xie-Xin-Tang (SHXXT), composed of three herbs, Radix et Rhizoma Rhei (*Rheum palmatum* L.), Radix scutellaria (*Scutellaria baicalensis* Georgi), and Rhizoma coptidis (*Coptis chinensis* Franch), was shown to be involved in attenuating inflammation of the airways, colon, and blood vessels [77]. The Banxia Xiexin Decoction (BXD), one of its active components being rhein, is prescribed to ease various inflammatory disorders like gastritis and upper airway inflammation [78]. The BXD was also effective in curing colon cancer in animals [79]. Additionally, studies demonstrated that the combination of cisplatin and the BXD decoction induced apoptosis in A549 human lung cancer cells [80]. A decoction of the herbs *Rheum palmatum* L., *Artemisia annua* L., and *Gardenia jasminoides* Ellis, popularly known as Yin-Chen-Hao-Tang (YCHT), is primarily used to treat several liver disorders. Pharmacokinetic studies have shown that the compounds rhein, geniposide and 6,7-dimethylesculetin, isolated from YCHT, enhanced the synergistic and therapeutic benefits, as demonstrated in animal models [81].

The Indian system of medicine Ayurveda suggests the use of *R. australe* (one of the sources of rhein), commonly found in the Himalayas, for curing multiple chronic diseases, including cancers of liver, breast and prostate [65]. The rhizomes of another species of *Rheum*, *R. emodi* showed antihelminthic, antiulcerative and anticancerous activities [82]. Its extract was also reported to be effective in curing *Helicobacter-pylori*-induced ulcers in animals [72]. Recent reports have demonstrated the antiinflammatory activity of anthraquinones found in *Cassia sp.* in the treatment of airway-associated allergies [83].

## 4. Chemistry of Rhein

The rhein molecule, or 4,5-dihydroxy-9,10-dioxoanthracene-2-carboxylic acid, is a planar compound with three fused benzene rings, has a molecular mass of 283.22 g/mol and the molecular formula C_15_H_8_O_6_ [84]. It is also popularly known as Rhubarb Yellow. Rhein is found in its free form or as glucosides in the Fabaceae and Polygonaceae family of plants [85]. This compound is water-insoluble, meaning it has low systemic bioavailability. However, the lipophilic nature of this compound permits it to easily get into cells [86]. As this compound has a highly stable structure, various hydrophilic and lipophilic nano-formulations have been developed to improve its oral absorption, bioavailability, and sustained targeted release [59]. A conjugate of rhein, rhein–DOTA (1,4,7,10-tetraazacyclododecane-1,4,7,10-tetraacetic acid) has been used to treat sarcoma and owing to its remarkable necrosis avidity; studies suggest that it could be used as a significant probe for PET/CT-imaging-mediated early detection of response to antitumor therapy [87].

The plasma concentration of rhein has also been compared with other anthraquinones found in traditional Chinese medicine, such as Rhei Rhizoma (used for treating various neuroinflammatory disorders and osteoarthritis) [88,89]. It was observed that there was a significant increase in serum concentrations of rhein, 12 hours after oral administration of Rhei Rhizoma, where the peak serum concentration was reported to be 126.50 ng/mL, which is comparably higher than rhein alone [90].

## 5. Biological Activities of Rhein

**Figure 2:**
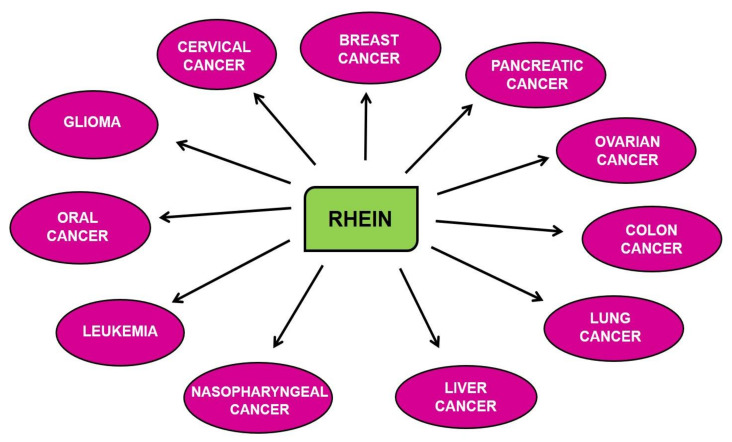
Anticancer activity of rhein in different cancers.

Rhein has been actively explored for its pharmacological benefits to human health. Many biological properties of rhein have been studied, such as curing inflammatory disorders like upper airway inflammation, asthma, gastritis, and fatty liver and protecting against cerebral ischemic injury, diabetic nephropathy, etc. [42,78,79,91,92]. Several studies have shown that rhein is an effective antidiabetic, antiosteoarthritic and anticathartic agent [39,40,44,45,60]. The antibacterial properties of rhein have been observed in the rhizomes of rhubarb (*R. officinale*) [93]. Some other studies have demonstrated that rhein has a higher antioxidative potential than another anthroquinone component of *P. multiflorum*, aloe-emodin. In addition, the chemiluminescent data for the free-radical-scavenging activity of rhein suggested that it is more effective than antioxidants like α-tocopherol and Vitamin C [94]. Rhein, and its derivatives and analogs, are known to show the anticancer activity against various cancers, as shown in Figure 2 and Table 1, which is of paramount interest.

## 6. Molecular Targets of Rhein

**Figure 3:**
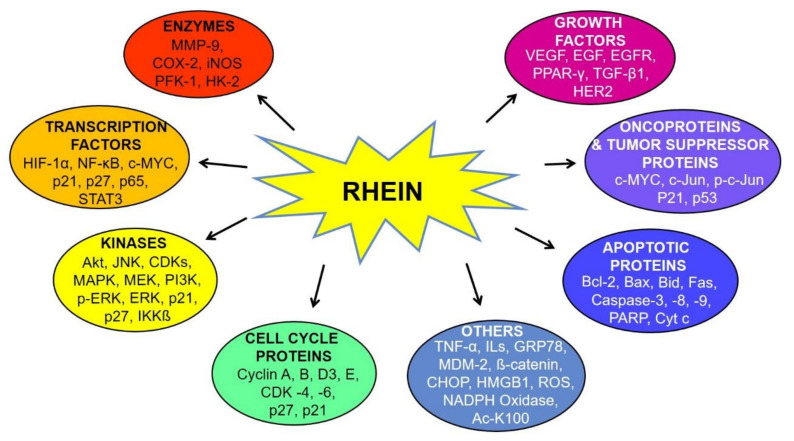
Molecular targets of rhein. Abbreviations: Ac-K100: acetylated lysine; Bax: Bcl-2-associated X protein; Bcl-2: B cell lymphoma 2; Bid: BH3 interacting domain death agonist; CDK: cyclin-dependent kinase; CHOP: CCAAT/enhancer-binding protein homologous protein; COX-2: cyclooxygenase 2; Cyt c: cytochrome c; EGF: extracellular growth factor; Fas: FS-7-associated surface antigen; GRP78: 78 kDa glucose regulated protein; HER-2: human epidermal growth factor receptor 2; HIF-1α: hypoxia-inducible factor 1 alpha; HK-2: hexokinase 2; HMGB1: high-mobility-group-box-1; IKKβ: inhibitor of nuclear factor kappa-B kinase subunit beta; IL: interleukin; iNOS: inducible nitric oxide synthase; JNK: c-Jun N-terminal kinase; MAPK: mitogen-activated protein kinase; MDM2: murine double minute-2; MEK: mitogen-activated protein kinase kinase; MMP-9: Matrix metalloproteinase-9; NADPH: nicotinamide adenine dinucleotide phosphate; NF-κB: nuclear factor kappa light chain enhancer of activated B cells; PARP: poly ADP ribose polymerase; p-c-Jun: phosphorylated c-Jun; pERK: phosphorylated extracellular signal-regulated kinase; PFK-1: phosphofructokinase-1; PI3K: phosphoinositide 3-kinase; PPAR-γ: peroxisome proliferator-activated receptor gamma; ROS: reactive oxygen species; STAT3: signal transducer and activator of transcription 3; TGF-β1: transforming growth factor beta 1; TNF-α: tumor necrosis factor alpha and VEGF: vascular endothelial growth factor.

Increasing lines of evidence suggest the anticancer activity of rhein against different cancers, as depicted in Figure 2 [43,46,54]. Rhein has been shown to inhibit the distinct hallmarks of cancer, including cell proliferation, angiogenesis, migration, epithelial to mesenchymal transition (EMT), etc. These cellular processes are regulated via the modulation of several cellular molecules such as enzymes, transcription factors, kinases, cell-cycle proteins, growth factors, oncoproteins, tumor suppressor proteins, apoptotic proteins, etc., as shown in Figure 3 [52,53,54,55,56]. Many important molecular pathways or proteins regulating the survival of cancerous cells are targets of rhein, including the sonic hedgehog pathway, serine-threonine kinases like Akt kinase, etc. [46]. Rhein is also known to exert its anti-inflammatory effect by modulation of nuclear factor—kappa light chain enhancer of activated B cells (NF-κB), which subsequently regulates the downstream nitric oxide synthase pathway [95]. The other important nuclear targets of this phytochemical include p53 and p21/WAF proteins, which aid in the induction of apoptosis [96]. The rhein-induced mitochondrial apoptotic pathway is activated by increased levels of Fas, cleaved caspases-3, -8, -9, poly(ADP-ribose) polymerase (PARP), etc. and decreased expression of B cell lymphoma 2 (Bcl-2), cyclin A and cyclin-dependant kinases (CDK) [97]. The antitumorigenic effects of this compound in ovarian cancer cells is exerted through inhibition of the phosphorylation of mitogen-activated protein kinase (MAPK) pathway elements like mitogen-activated protein kinase kinase (MEK) and extracellular-signal-regulated kinase (ERK) [47]. Under hypoxic conditions, rhein has also been shown to enhance cytotoxicity in colorectal cancer cells (CRC) by modulating the expression of hypoxia-inducible factor-1 alpha (HIF-1α) expression, which acts on immunosuppressive molecules such as the downstream elements programmed cell death ligand-1 (PD-L1), vascular endothelial growth factor (VEGF), cyclooxygenase 2 (COX-2) and galectin-1 [98]. The different pathways modulated by rhein are discussed below.

### 6.1. MAPK Signaling Pathway

The MAPK signaling pathway plays a vital role in cell proliferation and survival. These molecules influence the group of responsive enzymes recruited to deal with cellular stress caused by heat, osmosis, cytokines and ultraviolet (UV) irradiation [99]. The MAPK family of proteins are also involved in the generation of mitogenic responses and the production of stress response proteins in different cells of the body. The three major MAPK families are ERKs, Jun amino-terminal kinases (JNKs) and stress-activated protein kinases (p38/SAPKs) [100,101,102,103,104]. A recent study showed that HeLa cervical cancer cells underwent apoptosis upon modulation of the MAPK pathway by rhein. It was demonstrated that due to the binding of rhein lysinate (RHL or a salt of rhein and lysine), the phosphorylation of ERK1/2, JNK and p38 MAPK was enhanced, which activated growth-inhibitory pathways including the regulation of apoptotic proteins like increased levels of cleaved caspase-3/7 and PARP [48].

### 6.2. Wnt Signaling Pathway

As discussed earlier, rhein can inhibit cell proliferation in various cancer cells by targeting β-catenin, PI3K/Akt, ERK, p38 MAPK, JNK and fat mass and obesity-associated genes (FTO). Cyclin D1, one of the major cell-cycle mediator proteins, is overexpressed in the cancer cells due to the increased β-catenin levels. Thus, there is a crucial role of β-catenin, a component of the Wnt signaling pathway, in activating the genes for growth regulation involving cellular survival, proliferation and metastasis [105,106,107,108,109]. It was shown that rhein induced cell-cycle arrest at the G0/G1 and S phases in A549 lung cancer cells and BEL-7402 hepatocellular cancer cells, respectively [54,110]. It was demonstrated that rhein induced apoptosis in A549 cells due to the enhanced levels of GRP78 and reduced CDK-4, -6 and cyclin E (some of the molecular components of the Wnt pathway) [110]. Studies also showed that rhein suppressed the active levels of β-catenin in HepG2 human liver cancer cells and HeLa cervical cancer cells, which resulted in a cell-cycle arrest at the S phase [111]. Similarly, rhein induced cell-cycle arrest at the S-phase in BEL-7402 cells. The suppression of c-Myc, a target of the Wnt/β-catenin pathway, and the induction of caspase-3 by rhein suppressed the proliferation and survival of these cells [54].

### 6.3. NF-κB Signaling Pathway

One of the most rapid-acting transcription factors mediating inflammatory processes is nuclear factor-kappa B (NF-κB) [112,113,114,115,116,117]. It is kept inactive in the cell by the binding of IκB. Harmful stimuli like stress, UV, bacterial or viral antigens, cytokines, or free radicals, reactive oxygen species (ROS) cause IκB kinases to phosphorylate the IκB protein that translocates NF-κB to the nucleus for the transcription of different genes involved in inflammation and cancer. Therefore, active NF-κB leads to stimulation of an inflammatory response or immune response and promotes cell survival and cell proliferation [118,119,120,121,122,123,124]. Thus, putting a check on the activation of NF-κB and its accessory pathways can lead to the suppression of inflammation, angiogenesis, and carcinogenesis [62,125,126,127,128,129,130,131,132,133]. Studies have defined the role of many natural inhibitors of NF-κB that are obtained from the diet, which reduce inflammation and restore energy balance in humans [29,134]. This safe, well-tolerated natural compound was shown to inhibit LPS-induced NF-κB activation and regulatory pathways in RAW 264.7 macrophages by inhibiting the protein, inhibitor of nuclear factor kappa-B kinase subunit beta (IKKβ). It was also observed that rhein induced an antiinflammatory effect by modulating the expression of NF-κB and its downstream elements such as intracellular ROS, inducible nitric oxide synthase (iNOS), interleukin (IL)-6, and pro-inflammatory factors like IL-1β and high-mobility-group-box-1 (HMGB1) [135].

### 6.4. HIF-1 Signaling Pathway

Studies on the exposure of tumor cells to hypoxic conditions have shown enhanced levels of COX-2, PD-L1, IL-10, VEGF, galectin-1, and transforming growth factor-β1 (TGF-β1) [136]. These immunosuppressing molecules stop immune cell differentiation, drive apoptosis of T cells, and inhibit the development of dendritic cells. The transcription of these immunosuppressive molecules is driven by HIF-1α, which is produced due to hypoxia in tumor cells [137]. In a recent study involving breast cancer cells MCF-7 and MDA-MB-435, rhein was reported to play an important role in reducing tumor growth and vasculogenesis by inhibiting the expression of HIF-1α. The effect of rhein was also observed in hypoxia-induced angiogenesis in these cells, where HIF-1α and VEGF levels were reduced [138].

### 6.5. Other Signaling Pathways Regulated by Rhein

Matrix metalloproteinases (MMPs), a family of zinc proteases, help cancer cells to invade by degrading the extracellular matrix and the basal membrane [139]. They disrupt the structure of healthy tissues and thereby enhance disease progression. The MAPK family of proteins are modulated by ROS-mediated MMP activation to help in the invasion of tumors [2]. Gastric cancer cells have increased expression of upstream regulators NF-κB and activator protein (AP)-1 that direct the MMP gene activity. Studies have shown that rhein may act by suppressing JNK1/2 or p38 to modulate MMP through AP-1 expression [53]. Ser/Thr–Pro regulated protein phosphorylation is involved in epithelial cell proliferation and transformation [140]. The Pin1-driven Ser/Thr–Pro can activate oncoproteins like NF-κB and AP-1 and also destabilize tumor-suppressor genes like p53 by phosphorylation of Ser/Thr–Pro. Thus, the regulation of these proteins by rhein can provide a therapeutic target to kill cancer cells. It has been shown that the suppression of Pin1 in cancer cells leads to apoptosis or suppression of expression of the onco-proteins [141]. Studies have shown that rhein also targets the formation of the Pin1/c-Jun complex, which is an essential regulator of the cyclin D1 gene in the G2/M phase of cell-cycle progression. The disruption of pc-Jun (Ser73) and Pin1 bond was induced by rhein, which ultimately led to the cell cycle arrest in G2/M phase [142].

The pathways that induce cell death by apoptosis include two regulatory pathways. One pathway involves death-receptor-mediated caspase-8 activation, which can stimulate downstream caspase-3. Another pathway that induces apoptosis is mitochondria-mediated. The release of cytochrome c (Cyt c) from mitochondria leads to the activation of procaspase-9, which initiates an apoptosis-symbolic apoptosome formation composed of dATP (deoxyadenosine triphosphate), Apaf-1, procaspase-9 and Cyt c [143]. Apoptosome formation causes the activation of caspase-3, -6 and -7. Several studies have shown that rhein increases intracellular levels of nitric oxide, ROS, and Ca^2+^, stimulates apoptosis, and inhibits cell proliferation and angiogenesis both in vitro and in vivo [143]. Bim, a pro-apoptotic Bcl-2-family protein, is a critical mediator of rhein-induced apoptosis. Rhein activated forkhead box O3a (FOXO3a), an inducer of Bim expression, in MCF-7 and HepG2 cells, which in turn enhanced the Bim protein levels [144]. Elevated levels of ROS modulated apoptosis by affecting the mitochondrial membrane permeability (MMPE) and causing subsequent loss of membrane potential (ΔΨm) [43]. Some other studies have shown the role of MMPE in apoptosis induction, triggering the release of Cyt c [145]. Therefore, studies have demonstrated the apoptotic effect of rhein via modulation of ROS, MMPE, and caspases-3, -8, and -9, in SCC-4, A549 and HL-60 cancer cells [43,110,145].

**Figure 4:**
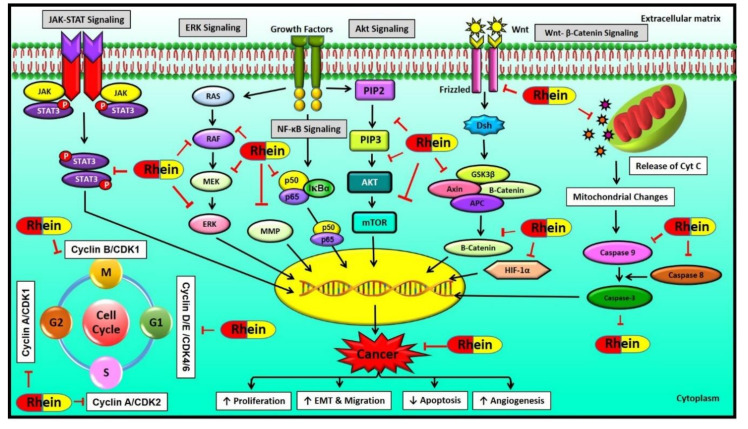
Mechanism of action of rhein. Abbreviations: APC: adenomatous polyposis coli; CDK: cyclin-dependent kinase; Dsh: dishevelled; EMT: epithelial to mesenchymal transition; ERK: extracellular signal-regulated kinase; GSK3β: glycogen synthase kinase 3β; HIF-1α: hypoxia-inducible factor 1 alpha; JAK: Janus kinase; MEK: mitogen-activated protein kinase kinase; MMP: matrix metalloproteinase; mTOR: mammalian target of rapamycin; NF-κB: nuclear factor kappa light chain enhancer of activated B cells; PIP2: phosphatidylinositol 4,5-bisphosphate; PIP3: phosphatidylinositol (3,4,5)-trisphosphate; and STAT: signal transducer and activator of transcription.

Rhein, being a planar molecule, has a significant structural advantage of being easily intercalated into the DNA molecule. Thus, an increase in DNA length consequently disrupts gene function. Rhein induced the DNA damage in targeted cancerous cells by inducing the apoptotic pathways that led to cell-cycle arrest [43]. Thus, rhein is a multitargeted compound, and its plausible mechanism of action, as evidenced by several studies, is shown in Figure 4.

## 7. Chemopreventive and Therapeutic Properties of Rhein for Different Cancers

Mounting evidence shows that the multitargeted compound rhein is minimally toxic, affordable and effective for the prevention and treatment of different types of cancers, as listed in Table 1. The following part of the review enumerates the anticancer potential of rhein for different cancers.

### 7.1. Breast Cancer

Breast cancer is the most commonly occurring cancer and leading cause of mortality in females worldwide [1,107,161,162,163,164,165]. A number of studies have been carried out to investigate the role of rhein in the prevention and treatment of breast cancer both in in vitro and in vivo. This compound suppressed proliferation and inhibited breast cancer in mice. For instance, the administration of rhein has been shown to suppress tumor growth in 4T1-cell-induced mouse xenografts [146]. This study also showed that rhein in combination with atezolizumab synergistically elevated the expression of apoptotic protein such as Bax and caspases-3, -8, and -9, and decreased that of Bcl-2 [146]. In another study, it was reported that rhein inhibited the phosphorylation of Akt and activated FOXO3a and further stimulated the activity of the pro-apoptotic protein Bim, which led to the cleavage of caspase proteins and subsequent induction of apoptosis in MCF-7 cells [144]. Rhein was also shown to inhibit NF-κB activation and its downstream targets HIF-1α and VEGF165 in breast cancer cells, MCF-7 and MDA-MB-435 [138]. In addition, rhein was shown to inhibit phosphorylation of HER-2 protein in SK-Br-3 cells in vitro, thus showing its potential in the development of therapies for HER-2-positive breast cancer [147]. Not only rhein, but also its derivatives showed promising effects in breast cancer. For example, a novel rhein-derived compound, 4F, was found to induce autophagy in MDA-MB-231 cells by upregulating the expression of beclin-1 and causing the degradation of p62 [148]. Furthermore, it was observed that an analog of rhein, rhein lysinate (RHL), inhibited phosphorylation of EGFR, MEK, c-Raf, and ERK and induced apoptosis in MCF-7, SK-Br-3, and MDA-MB-231 cells [46]. This study also demonstrated that rhein has the potential to sensitize breast cancer cells to taxol by decreasing the levels of phospho-epidermal growth factor receptor (p-EGFR), thus unravelling the potential of this anthraquinone for the management of drug resistance in breast cancer cells [46].

### 7.2. Cervical Cancer

Globally, cervical cancer is one of the leading causes of cancer-related deaths among women, with annual incidence rates of 500,000 patients [1,166]. Several studies have showcased the anticancer potential of rhein in cervical cancer. For instance, rhein was shown to inhibit β-catenin and c-Myc, which are highly overexpressed in cervical cancer cells, by suppressing the phosphorylation of GSK3β and inducing S-phase cell-cycle arrest in HeLa cells [111]. In another study, Ip et al. (2007) showed that in Ca Ski cervical cancer cells, rhein induced apoptosis via the mitochondrial pathway. It was observed that treatment with rhein induced Ca^2+^ release from the endoplasmic reticulum, which was followed by disruption of the mitochondrial membrane potential due to the release of Cyt c, activated caspase-3 and PARP cleavage, leading to apoptosis in these cells [149]. The rhein derivative RHL was also found to inhibit the proliferation of HeLa cells in a dose- and time-dependent manner through the phosphorylation and activation of the downstream regulators of MAPKs such as JNK, p38 MAPK and ERK1/2, thereby inducing the activation of apoptotic proteins like cleaved caspase-3/7 and PARP [48].

### 7.3. Colon Cancer

Colon cancer is the third leading cause of cancer-related deaths worldwide. The anticancer potential of rhein against colon cancer has been reported by both in vitro and in vivo studies. For example, rhein has been shown to inhibit the proliferation of Caco-2 cells by modulating the MAPK pathway [150]. It was also observed that rhein induced apoptosis in HCT-116 and SW620 cells by suppressing phospho-signal transducer and activator of transcription 3 (p-STAT3), Bcl-2, cyclin D1, and cyclin B1 [151]. Moreover, in HT29 cells, rhein showed a suppressive activity over the elevated expression of HIF-1α and several immunosuppressive molecules like PD-L1, VEGF, COX-2, IL-10, etc., that were found to be involved in the survival of these cancer cells. [98]. Although rhein showed significant cytotoxicity in colon cancer cell line COLO 320 DM, it was found to be safe and did not cause DNA damage to normal colon cells, thus exhibiting selective toxicity [69]. In addition, recent studies on animal models of colon cancer have shown possible involvement of rhein in alleviating tumor proliferation via the activation of MAPK/NF-κB pathways [79].

### 7.4. Glioma

Glioma and glioblastoma are some of the severe forms of rapidly developing brain tumors [7,153]. Rhein and its derivatives have shown significant effect in suppressing the proliferation and survival of these cancer cells. For example, the treatment of rhein suppressed the activation of ERK1/2 by and elevated Bcl-2 and caspase-3 levels, induced apoptosis and cell-cycle arrest in rat F98 (sub-S phase arrest) and human Hs683 (G2/M phase arrest) glioma cell lines [152]. In another study, a novel rhein-derived compound, hydroxyethyl hydroxamic acid (SYSUP007), was found to suppress the proliferation, invasion, and migration of glioblastoma cells T98G and U251. Additionally, the treatment of glioblastoma cells with this formulation was shown to inhibit MMP-9 and SGK-1, which are involved in drug resistance and tumor development, and also this formulation induced the expression of Ac-K100 (acetylated lysine) in these cells [153].

### 7.5. Leukemia

Mutation in immature lymphocytes leads to uncontrolled or non-functional development of the derived blood cells. These cancerous cells in lymphoblasts or acute lymphoblastic leukemia (ALL) are the cause of 25% of all childhood cancers [167]. The antileukemic effect of rhein was studied on the acute promyelocytic leukemia cell lines NB4 and HL-60 [146,163]. Rhein was shown to target the Bid protein, which caused the loss of mitochondrial potential due to Cyt c efflux and induced the cleavage of an apoptotic executioner caspase, caspase-3, in HL-60 cells [145]. Besides the involvement of mitochondrial and caspase pathway, studies have shown that rhein also targets the signalling complexes p-ERK, PI3K, and p-Akt, which leads to the apoptosis of human NB4 cells [155]. In another study, the rhein-derived compound AQ-101 was shown to disrupt MDM2 protein interaction and controlled the proliferation of ALL cells in vitro and in vivo by modulating the expression of p53 [154].

### 7.6. Liver Cancer

Liver cancer is the second leading cause of cancer-related deaths and the sixth-most common form of cancer in the world [168,169,170,171,172,173,174,175,176]. The anticancerous activities of rhein have been well studied in liver cancer. An in vitro study on the effect of rhein against HepaRG cells showed that it induced cell cycle arrest at S phase by modulating the expression of cyclin E, p53, p21, cyclin A, and CDK-2 [97]. Another study on the effect of rhein against arrested Hep G2 cells revealed that rhein arrested cell growth in the G1 phase of the cell cycle by inducing p53 and p21/WAF1 proteins [96]. Recent studies have shown that this compound induces apoptosis and G0/G1 cell-cycle arrest via regulation of ROS in both HepG2 and Huh7 cells [55].

As an essential apoptotic inducer, the Bim protein plays a vital role in inducing angiogenesis and tumorigenesis in cancer cells. Rhein-mediated modulation of phosphorylation of Akt and FOXO3a induced Bim secretion and caused apoptosis in HepG2 cells [144]. It was shown that rhein also incited the release of mitochondrial Cyt c and hindered the synthesis of ATP, which induced apoptosis due to the loss of mitochondrial membrane potential in HepaRG cells and SMMC-7721/DOX cells, respectively [97,156]. In another study, it was reported that the proliferation of hepatocellular cells, BEL-7402 was significantly decreased by rhein via regulation of caspase-3 and oncogene c-Myc [54]. The anticancer effect of rhein was also studied in in vivo models of liver cancer. For example, rhein was shown to inhibit HepG2-induced xenograft tumorigenesis in animals, by suppressing β-catenin expression and GSK3β levels to reduce the tumor growth. This antiproliferative activity of rhein on cell proliferation of HepG2 liver cancer cells was also due to the decrease of β-catenin, induced by modulation of p53 and K-ras proteins [111].

### 7.7. Lung Cancer

Lung cancer is the most diagnosed cancer in the world [1,177,178,179,180,181,182,183,184]. Many reports have also highlighted the potential therapeutic effects of rhein against lung cancer. Bu et al. (2020) demonstrated that rhein induced apoptosis in A549 cells by modulating the expression of p-AMPK, mTOR, and Bcl-2 proteins [158]. The reduced Bcl-2 expression, Cyt c efflux, the rapid loss of ΔΨm and enhanced caspase-3 activity led to apoptosis in these cells. Moreover, treatment with rhein also resulted in increased expression levels of p53 and p21 and decreased levels of CDK-4 and -6 and cyclin E, which led to G0/G1 cell-cycle arrest [110]. Some preclinical studies also showed the regulation of STAT3, Bax, and Bcl-2 expression pathway by rhein in A549 cells. It was shown that the treatment of rhein halted the cell cycle at G2/M phase via downregulation of cyclin B1, MDM2, and p-53 expression levels in H460 and PC-9 cells [157].

### 7.8. Nasopharyngeal Cancer (NPC)

The anticancer activity of rhein has also been reported in NPC, a rare form of head and neck cancer. It was shown that treatment with rhein modulated the activating transcription factor (ATF)6 and p-ERK-regulated induction of endoplasmic reticulum (ER) stress-associated apoptosis factor and CCAAT/enhancer-binding protein homologous protein (CHOP) in a dose- and time-dependent manner. The observed changes in the levels of cytosolic Ca^2+^, ROS, and MMP had an antiproliferative impact in these cells. It was also shown that rhein modulated the activities of caspase-8 and -9 via the activation of CHOP and caspase-3 [49].

### 7.9. Ovarian Cancer

Ovarian cancer is a cancer that develops in the ovaries. Rac1, a small G protein family member, influences cell migration and invasion by generating ROS in these cancer cells. Rhein was shown to reduce the activity of Rac1 and AP-1, thereby regulating its downstream ROS-dependent signaling axis p38/JNK MAPK in SKOV3-PM4 cells, and attenuating cellular proliferation, migration, and invasion [53]. Rhein also inhibited matrix metalloproteinases in A2780 and OV2008 cell lines [159].

### 7.10. Pancreatic Cancer (PC)

Cancers of the pancreas are some of the most lethal forms of cancer [64]. Rhein, along with EGFR inhibitors, was shown to inhibit STAT3 in PC. The combination of rhein along with the EGFR inhibitor erlotinib against PANC-1 and BxPC-3 xenograft mouse models displayed a synergistic effect by diminishing the expression of p-STAT3 and p-EGFR. Additionally, it also inhibited Bcl-2 levels and enhanced Bax levels in the in vivo models [52]. Rhein was also shown to counter the Warburg effect in MiaPaCa2 cells. The preclinical studies suggested that there was a decrease in levels of HIF-α and associated proteins and PFK (phosphofructokinase)-1 after the treatment with rhein. Thus, rhein helped to improve the glucose homeostasis in these cancer cells [160].

### 7.11. Oral Cancer

Cancers of the oral cavity is a major health concern worldwide [1]. Few studies have shown the efficacy of rhein against oral cancer. Rhein was reported to have an essential role in preventing the migration and invasion of SCC-4 cells by suppressing the activity of the MMP-9 gene [50]. It led to DNA damage in these cells via inhibition of mRNA expression of DNA-repair-associated genes like O(6)-methylguanine-DNA methyltransferase (MGMT) [51]. Moreover, the compound also induced S-phase arrest in SCC-4 cells by inhibiting cyclin B1 and cyclin A in vitro. In addition, there was an induction of caspase-3 proteases and reduction in Bcl-2 levels in the cells treated with rhein. It was also seen that ROS and Ca^+2^ levels could be involved in rhein-induced apoptosis [43].

## 8. Toxicity of Rhein

Toxicity is often associated with the use of drugs and medicines, and remains a major challenge related to drug safety and clinical applications. The toxicity of rhein has been reported in a few studies. For example, Xianghong et al. (2010) showed the acute hepatotoxicity associated with the plant extracts of *P. multiforum* in mice, which was due to one of its anthraquinone components, rhein [185]. Rhein also induced toxicity at the dosage of 6.25–50 μM in hepatocytes in vitro [185]. In another investigation, it was observed that rhein induced apoptosis in renal epithelial HK-2 cells through the uncoupling protein (UCP)-2-related mitochondrial pathway [186]. In addition, the dose-dependent effects of rhein exerted a toxic effect in hepatic HL-7702 cells via the oxidative stress-involved mitochondria-mediated apoptotic pathway. After 12 hours of treatment, a significant apoptotic effect was observed at 50 μM and 100 μM concentrations of rhein [187]. However, in most of the studies, it was observed that very high concentrations of rhein induced toxicity in normal cells.

## 9. Conclusions

Rhein, isolated from rhubarb herbs, an ancient Chinese medicine, has been in use for thousands of years. In developing parts of world like India and Africa, around 65% and 80% of the population, respectively, make use of phytomedicines. To obtain the maximum benefit from these multitargeted phytochemicals in the prevention and treatment of chronic diseases, they have been used in conjunction with monotargeted modern medicines [188,189,190,191,192]. Rhein was shown to induce the anticancerous effect through multiple mechanisms. This compound rhein has the potential to modulate various key regulatory pathways like NF-κB, PI3K/Akt, MAPKs, etc., which are important for the regulation of several chronic diseases like arthritis, renal dysfunction, neuronal dysfunction, etc. There are many more unidentified cellular molecules that interact with rhein, which are involved in inducing cytotoxicity in the cancer cells. The research into its potential effects with other established anticancer drugs and nanomatrix formulation is still in its infancy. This novel compound lacks momentum in research due to its high hydrophobicity which leads to its low bioavailability. Thus, with improvement in its physicochemical dynamics, this multimodal molecule could be of more value to human health.

With the discovery of induction routes of cancer, the race to identify a cure has accelerated [90]. To translate phytochemicals into clinical setting, the efficiency of the drugs needs to be improved through enhancing the drug interactions, drug dynamics, and other pharmacokinetic parameters [193]. Although the biological effect of *Rheum* rhizomes in several polyherbal extracts are of high ethnopharmacological importance, there are fewer data available from pharmacokinetic studies of rhein alone. As the source of rhein is botanical, it could significantly decrease the expenses involved in treating cancer. Apart from the need to conduct more in vitro and in vivo studies, clinical trials have to be performed with rhein to validate its anticancerous therapeutic potential. Prior to clinical trials with this compound, it will be necessary to identify the safe levels of this compound with regard to active blood levels, retention ability, and disposed metabolites [90]. Thus, this novel compound could be of high value to human health when used at appropriate concentrations and in the form of formulations with enriched bioavailability and upgraded pharmacodynamics.

## Figures and Tables

**Table 1:** Chemopreventive activity of rhein in different cancers.

Cancer	In Vitro/In Vivo /Ex Vivo	Model	Mechanism of Action	References
Breast cancer	In vivo	4T1 xenograft mice	Caspase-3, -8, -9↑, TNF-α↑, IL-6↑	[146]
	In vitro and in vivo	MCF-7, SK-Br-3, and MDA-MB-231 cells	p-EGFR↓, p-ΜΕΚ↓, p-ERK↓	[46]
		MCF-7 injected BALB/c athymic mice		
	In vitro	SK-Br-3	p-HER-2↓, NF-κB↓, p53↑, p21↑	[147]
	In vitro	MCF-7	Cleaved caspase↑, p-Akt↓, FOXO3a↑, Bim↑	[144]
	In vitro	MCF-7, MDA-MB-435s	PI3K↓, p-Akt↓, p-ERK↓, NF-κB↓, HIF-1α↓, EGF↓	[138]
			Hsp90α↓, COX-2↓, HER-2↓, VEGF(165)↓, p-I-κB↓	
	In vitro	MDA-MB-231	Beclin-1↑, LC3-II/LC3-I↑, p62↓	[148]
Cervical cancer	In vitro	HeLa	MAPK↑, JNK↑, p-ERK↑,	[48]
			cleaved PARP↑, Caspase-3, -7↑	
	In vitro	HeLa	β-catenin↓, S phase arrest↑	[111]
	In vitro	CaSki	Cytc↑, Caspase-3, -8, -9↑, Fas↑, p53↑, p21↑, Bcl-2↓,	[149]
			ΔΨm↓, cleaved Bid↑, cleaved PARP↑	
Colon cancer	In vitro	Caco-2	p-ERK1/2↑ (at higher concentrations of rhein)	[150]
	In vitro	HT29, HCT116, Colo205, SW620	HIF-1α↓, PD-L1↓, VEGF↓, COX-2↓, galectin-1↓	[98]
	In vitro	HCT116, SW620	p-STAT3↓	[151]
Glioma	In vitro	F98	ERK1/2↓	[152]
	In vitro	T98G, U87, U251	Ac-K100↑, NDRG1↑	[153]
Leukemia	In vivo	EU-1 injected SCID mice	MDM2↓, p53↑	[154]
	In vitro	HL-60	Cleaved caspase↑, cleaved PARP↑, cleaved Bid↑, ΔΨm↓	[145]
	In vitro	NB4	p-ERK↑, Caspase-3↑	[155]
Liver cancer	In vitro and in vivo	HepG2, HepG2 injected BALB/c-nu mice	β-catenin↓, S phase arrest↑	[111]
	In vitro	HepG2	CD95↑, p53↑, p21/WAF↑, mCD95L↑, sCD95L↑	[96]
	In vitro	BEL-7402	c-Myc↓, Caspase-3↑, S phase arrest↑	[54]
	In vitro	HepG2	p-Akt↓, FOXO↑, Bim↑, CHOP↑, p-eIF2α↑, p-ERK↓,	[144]
			Caspase-3, -8, -9↑	
	In vitro	HepaRG	ROS↑, ΔΨm↓, Bcl-2↓, Cyclin A↓, S-phase arrest↑	[97]
	In vitro	SMMC-7721, SMMC-7721/DOX	ATP synthesis↓, inner ΔΨm↓	[156]
	In vitro	HepG2, Huh7	ROS↑, p-c-Jun↑, Caspase-3↑	[55]
Lung cancer	In vitro and in vivo	PC-9, H460, A549, H460 xenograft mice	STAT3↓, Bax↑, Bcl-2↓, G2/M phase arrest↑	[157]
	In vitro	A549	p-PI3K↓, Akt↓, mTOR↓, Bcl-2↓	[158]
	In vitro	A549	G0/G1 phase arrest↑, GADD153↑, GRP78↑, Cyt c↑,	[110]
			Caspase-8↑, Bax↑,Bcl-2↓, Cleaved Bid↑, Cyclin D3↓,	
			Cyclin E↓, CDK-4↓, CDK-6↓, ROS↑, p53↑, p21↑, ΔΨm↓	
Nasopharyngeal cancer	In vitro	NPC	GRP78↑, ATF6↑, CHOP↑, ROS↑,	[49]
			Caspase-3, -8,-9↑	
Ovarian cancer	In vitro	SKOV3-PM4	Rac1↓, ROS↓, MAPK↓,	[53]
			TIMP-1↑, TIMP-2↑,AP-1↓	
	In vitro	A2780, OV2008	MMP↓	[159]
Pancreatic cancer	In vitro and in vivo	AsPC-1, Patu8988T,	p-STAT3↓	[52]
		BxPC-3,PANC-1 injected BALB/c athymic mice		
	In vitro and in vivo	AsPC-1, BxPC-3, HPAF-2, MiaPaCa2, Panc-1,	HIF-1α↓, PFK-1↓, HK-II↓, Glut-1↓	[160]
		MiaPaCa2 injected athymic Balb/c mice		
Oral cancer	In vivo	SCC-4	p53↓, cyclin A & E↓, ER Ca^2+^↑, ROS↑,	[43]
			Caspase-3, -8, -9↑, Bcl-2↓, Cyt c↑	
	In vitro	SCC-4	MMP-9↓	[50]

↑: Upregulated; ↓: Downregulated.

## Abbreviations

Ac-K100	Acetylated lysine
ATF6	Activating transcription factor 6
ATP	Adenosine triphosphate
Bax	Bcl-2-associated X protein
Bcl-2	B cell lymphoma 2
Bid	BH3 interacting domain death agonist
CD	Cluster of differentiation
c-PARP	cleaved- Poly ADP ribose polymerase
CHOP	CCAAT/enhancer-binding protein homologous protein
COX-2	Cyclooxygenase 2
EGFR	Epidermal growth factor receptor
ER	Endoplasmic reticulum
ERK	Extracellular signal-regulated kinase
FOXO	Class O of Forkhead box transcription factors
FOXO3a	Forkhead box O3a
Fas	FS-7-associated surface antigen
GADD153	Growth arrest and DNA damage153
Glut-1	Glucose transporter1
GRP78	78 kDa Glucose-regulated protein
HER-2	Human epidermal growth factor receptor 2
HIF-1α	Hypoxia-inducible factor 1 alpha
HK-II	Hexokinase2
HMGB1	High-mobility-group-box-1
IL-6	Interleukin-6
IL-1β	Interleukin-1 beta
IKKβ	Inhibitor of nuclear factor kappa –B kinase subunit beta
JNK	c-Jun N-terminal kinase
mTOR	Mammalian target of rapamycin
MAPK	Mitogen-activated protein kinase
mCD95L	Membrane-bound CD95 ligand
MDM2	Murine double minute 2
MEK	Mitogen-activated protein kinase kinase
MMP	Matrix metalloproteinase
NO	Nitric oxide
NADPH oxidase	Nicotinamide adenine dinucleotide phosphate oxidase
NDRG1	N-myc downstream regulated 1
NF-κB	Nuclear factor- kappa light chain enhancer of activated B cells
p-Akt	Phosphorylated Akt
PARP	Poly ADP ribose polymerase
PFK-1	Phosphofructokinase-1
p-HER-2	Phosphorylated human epidermal growth factor receptor 2
p-EGFR	Phosphorylated epidermal growth factor receptor
p-c-Jun	Phosphorylated c-Jun.
p-eIF2α	Phosphorylated eukaryotic initiation factor 2 alpha
RAC1	Ras-related C3 botulinum toxin substrate 1
ROS	Reactive oxygen species
sCD95L	Soluble CD95 ligand
STAT3	Signal transducer and activator of transcription 3
TIMP	Tissue inhibitor of metalloproteinase
TNF-α	Tumor necrosis factor- alpha
UPR	Unfolded protein response
VEGF	Vascular endothelial growth factor

## References

[1] Bray F., Ferlay J., Soerjomataram I., Siegel R.L., Torre L.A., Jemal A. (2018). Global cancer statistics 2018: GLOBOCAN estimates of incidence and mortality worldwide for 36 cancers in 185 countries. CA A Cancer J. Clin..

[2] Devi Khwairakpam A., Monisha J., Roy N.K., Bordoloi D., Padmavathi G., Banik K., Khatoon E., Kunnumakkara A.B. (2019). Vietnamese coriander inhibits cell proliferation, survival and migration via suppression of Akt/mTOR pathway in oral squamous cell carcinoma. J. Basic Clin. Physiol. Pharmacol..

[3] Banik K., Harsha C., Bordoloi D., Lalduhsaki Sailo B., Sethi G., Leong H.C., Arfuso F., Mishra S., Wang L., Kumar A.P. (2018). Therapeutic potential of gambogic acid, a caged xanthone, to target cancer. Cancer Lett..

[4] Padmavathi G., Roy N.K., Bordoloi D., Arfuso F., Mishra S., Sethi G., Bishayee A., Kunnumakkara A.B. (2017). Butein in health and disease: A comprehensive review. Phytomed. Int. J. Phytother. Phytopharm..

[5] Roy N.K., Deka A., Bordoloi D., Mishra S., Kumar A.P., Sethi G., Kunnumakkara A.B. (2016). The potential role of boswellic acids in cancer prevention and treatment. Cancer Lett..

[6] Monisha J., Jaiswal A., Banik K., Choudhary H., Singh A.K., Bordoloi D., Kunnumakkara A.B. (2018). Cancer Cell Chemoresistance: A Prime Obstacle in Cancer Therapy. Cancer Cell Chemoresistance and Chemosensitization.

[7] Khwairakpam A.D., Monisha J., Banik K., Choudhary H., Sharma A., Bordoloi D., Kunnumakkara A.B. (2018). Chemoresistance in Brain Cancer and Different Chemosensitization Approaches. Cancer Cell Chemoresistance and Chemosensitization.

[8] Padmavathi G., Monisha J., Banik K., Thakur K.K., Choudhary H., Bordoloi D., Kunnumakkara A.B. (2018). Different chemosensitization approaches to overcome chemoresistance in prostate cancer. Cancer Cell Chemoresistance and Chemosensitization.

[9] Javadi M., Roy N.K., Sharma A., Banik K., Ganesan P., Bordoloi D., Kunnumakkara A.B. (2018). Chemoresistance and chemosensitization in Melanoma. Cancer Cell Chemoresistance and Chemosensitization.

[10] Chinese Pharmacopoeia Commission (2005). Pharmacopoeia of the People’s Republic of China.

[11] Kunnumakkara A.B., Banik K., Bordoloi D., Harsha C., Sailo B.L., Padmavathi G., Roy N.K., Gupta S.C., Aggarwal B.B. (2018). Googling the Guggul (Commiphora and Boswellia) for Prevention of Chronic Diseases. Front. Pharmacol..

[12] Khwairakpam A.D., Bordoloi D., Thakur K.K., Monisha J., Arfuso F., Sethi G., Mishra S., Kumar A.P., Kunnumakkara A.B. (2018). Possible use of Punica granatum (Pomegranate) in cancer therapy. Pharmacol. Res..

[13] Lu K., Zhang C., Wu W., Zhou M., Tang Y., Peng Y. (2015). Rhubarb extract has a protective role against radiation-induced brain injury and neuronal cell apoptosis. Mol. Med. Rep..

[14] Kashyap D., Tuli H.S., Yerer M.B., Sharma A., Sak K., Srivastava S., Pandey A., Garg V.K., Sethi G., Bishayee A. (2019). Natural product-based nanoformulations for cancer therapy: Opportunities and challenges. Semin. Cancer Biol..

[15] Merarchi M., Sethi G., Shanmugam M.K., Fan L., Arfuso F., Ahn K.S. (2019). Role of Natural Products in Modulating Histone Deacetylases in Cancer. Molecules.

[16] Shanmugam M.K., Warrier S., Kumar A.P., Sethi G., Arfuso F. (2017). Potential Role of Natural Compounds as Anti-Angiogenic Agents in Cancer. Curr. Vasc. Pharmacol..

[17] Tewari D., Nabavi S.F., Nabavi S.M., Sureda A., Farooqi A.A., Atanasov A.G., Vacca R.A., Sethi G., Bishayee A. (2018). Targeting activator protein 1 signaling pathway by bioactive natural agents: Possible therapeutic strategy for cancer prevention and intervention. Pharmacol. Res..

[18] Hsieh Y.S., Yang S.F., Sethi G., Hu D.N. (2015). Natural bioactives in cancer treatment and prevention. BioMed Res. Int..

[19] Yang S.F., Weng C.J., Sethi G., Hu D.N. (2013). Natural bioactives and phytochemicals serve in cancer treatment and prevention. Evid. Based Complementary Altern. Med. Ecam.

[20] Prasannan R., Kalesh K.A., Shanmugam M.K., Nachiyappan A., Ramachandran L., Nguyen A.H., Kumar A.P., Lakshmanan M., Ahn K.S., Sethi G. (2012). Key cell signaling pathways modulated by zerumbone: Role in the prevention and treatment of cancer. Biochem. Pharmacol..

[21] Ramachandran L., Manu K.A., Shanmugam M.K., Li F., Siveen K.S., Vali S., Kapoor S., Abbasi T., Surana R., Smoot D.T. (2012). Isorhamnetin inhibits proliferation and invasion and induces apoptosis through the modulation of peroxisome proliferator-activated receptor gamma activation pathway in gastric cancer. J. Biol. Chem..

[22] Yarla N.S., Bishayee A., Sethi G., Reddanna P., Kalle A.M., Dhananjaya B.L., Dowluru K.S., Chintala R., Duddukuri G.R. (2016). Targeting arachidonic acid pathway by natural products for cancer prevention and therapy. Semin. Cancer Biol..

[23] Thakur K.K., Bordoloi D., Prakash J., Javadi M., Roy N.K., Kunnumakkara A.B. (2018). Different Chemosensitization Approaches for the Effective Management of HNSCC. Cancer Cell Chemoresistance and Chemosensitization.

[24] Padmavathi G., Bordoloi D., Banik K., Javadi M., Singh A.K., Kunnumakkara A.B. (2018). Mechanism of Chemoresistance in Bone Cancer and Different Chemosensitization Approaches. Cancer Cell Chemoresistance and Chemosensitization.

[25] Bordoloi D., Monisha J., Roy N.K., Padmavathi G., Banik K., Harsha C., Wang H., Kumar A.P., Arfuso F., Kunnumakkara A.B. (2019). An Investigation on the Therapeutic Potential of Butein, A Tretrahydroxychalcone Against Human Oral Squamous Cell Carcinoma. Asian Pac. J. Cancer Prev. APJCP.

[26] Girisa S., Shabnam B., Monisha J., Fan L., Halim C.E., Arfuso F., Ahn K.S., Sethi G., Kunnumakkara A.B. (2019). Potential of Zerumbone as an Anti-Cancer Agent. Molecules.

[27] Sailo B.L., Banik K., Padmavathi G., Javadi M., Bordoloi D., Kunnumakkara A.B. (2018). Tocotrienols: The promising analogues of vitamin E for cancer therapeutics. Pharmacol. Res..

[28] Ranaware A.M., Banik K., Deshpande V., Padmavathi G., Roy N.K., Sethi G., Fan L., Kumar A.P., Kunnumakkara A.B. (2018). Magnolol: A Neolignan from the Magnolia Family for the Prevention and Treatment of Cancer. Int. J. Mol. Sci..

[29] Monisha J., Padmavathi G., Roy N.K., Deka A., Bordoloi D., Anip A., Kunnumakkara A.B. (2016). NF-kappaB Blockers Gifted by Mother Nature: Prospectives in Cancer Cell Chemosensitization. Curr. Pharm. Des..

[30] Padmavathi G., Rathnakaram S.R., Monisha J., Bordoloi D., Roy N.K., Kunnumakkara A.B. (2015). Potential of butein, a tetrahydroxychalcone to obliterate cancer. Phytomed. Int. J. Phytother. Phytopharm..

[31] Liskova A., Stefanicka P., Samec M., Smejkal K., Zubor P., Bielik T., Biskupska-Bodova K., Kwon T.K., Danko J., Büsselberg D. (2020). Dietary phytochemicals as the potential protectors against carcinogenesis and their role in cancer chemoprevention. Clin. Exp. Med..

[32] Kunnumakkara A.B., Sung B., Ravindran J., Diagaradjane P., Deorukhkar A., Dey S., Koca C., Yadav V.R., Tong Z., Gelovani J.G. (2010). γ-Tocotrienol inhibits pancreatic tumors and sensitizes them to gemcitabine treatment by modulating the inflammatory microenvironment. Cancer Res..

[33] Bordoloi D., Roy N.K., Monisha J., Padmavathi G., Kunnumakkara A.B. (2016). Multi-Targeted Agents in Cancer Cell Chemosensitization: What We Learnt from Curcumin Thus Far. Recent Pat. Anti-Cancer Drug Discov..

[34] Banik K., Ranaware A.M., Harsha C., Nitesh T., Girisa S., Deshpande V., Fan L., Nalawade S.P., Sethi G., Kunnumakkara A.B. (2020). Piceatannol: A natural stilbene for the prevention and treatment of cancer. Pharmacol. Res..

[35] Banik K., Sailo B.L., Thakur K.K., Jaiswal A., Monisha J., Bordoloi D., Kunnumakkara A.B. (2018). Potential of different chemosensitizers to overcome chemoresistance in cervical cancer. Cancer Cell Chemoresistance and Chemosensitization.

[36] Sailo B.L., Bordoloi D., Banik K., Khwairakpam A.D., Roy N.K., Prakash J., Kunnumakkara A.B. (2018). Therapeutic strategies for chemosensitization of renal cancer. Cancer Cell Chemoresistance and Chemosensitization.

[37] Sailo B.L., Javadi M., Jaiswal A., Prakash J., Roy N.K., Thakur K.K., Banik K., Bordoloi D., Kunnumakkara A.B. (2018). Molecular Alterations Involved in Pancreatic Cancer Chemoresistance and Chemosensitization Strategies. Cancer Cell Chemoresistance and Chemosensitization.

[38] Choudhary H., Thakur K.K., Sharma A., Roy N.K., Khwairakpam A.D., Bordoloi D., Kunnumakkara A.B. (2018). Strategies to Overcome Chemoresistance in Ovarian Cancer. Cancer Cell Chemoresistance and Chemosensitization.

[39] Mohammed A., Ibrahim M.A., Tajuddeen N., Aliyu A.B., Isah M.B. (2020). Antidiabetic potential of anthraquinones: A review. Phytother. Res..

[40] Zhou Y.X., Xia W., Yue W., Peng C., Rahman K., Zhang H. (2015). Rhein: A Review of Pharmacological Activities. Evid. Based Complementary Altern. Med..

[41] Zhang Y., Fan S., Hu N., Gu M., Chu C., Li Y., Lu X., Huang C. (2012). Rhein Reduces Fat Weight in db/db Mouse and Prevents Diet-Induced Obesity in C57Bl/6 Mouse through the Inhibition of PPARgamma Signaling. PPAR Res..

[42] Sheng X., Wang M., Lu M., Xi B., Sheng H., Zang Y.Q. (2011). Rhein ameliorates fatty liver disease through negative energy balance, hepatic lipogenic regulation, and immunomodulation in diet-induced obese mice. Am. J. Physiol. Endocrinol. Metab..

[43] Lai W.W., Yang J.S., Lai K.C., Kuo C.L., Hsu C.K., Wang C.K., Chang C.Y., Lin J.J., Tang N.Y., Chen P.Y. (2009). Rhein induced apoptosis through the endoplasmic reticulum stress, caspase- and mitochondria-dependent pathways in SCC-4 human tongue squamous cancer cells. In Vivo.

[44] Zheng J.M., Zhu J.M., Li L.S., Liu Z.H. (2008). Rhein reverses the diabetic phenotype of mesangial cells over-expressing the glucose transporter (GLUT1) by inhibiting the hexosamine pathway. Br. J. Pharmacol..

[45] Li H., Liang C., Chen Q., Yang Z. (2011). Rhein: A potential biological therapeutic drug for intervertebral disc degeneration. Med. Hypotheses.

[46] Lin Y.J., Zhen Y.S. (2009). Rhein lysinate suppresses the growth of breast cancer cells and potentiates the inhibitory effect of Taxol in athymic mice. Anti-Cancer Drugs.

[47] Lin Y.J., Zhen Y.Z., Shang B.Y., Zhen Y.S. (2009). Rhein lysinate suppresses the growth of tumor cells and increases the anti-tumor activity of Taxol in mice. Am. J. Chin. Med..

[48] Zhen Y.Z., Lin Y.J., Gao J.L., Zhao Y.F., Xu A.J. (2011). Rhein lysinate inhibits cell growth by modulating various mitogen-activated protein kinases in cervical cancer cells. Oncol. Lett..

[49] Lin M.L., Chen S.S., Lu Y.C., Liang R.Y., Ho Y.T., Yang C.Y., Chung J.G. (2007). Rhein induces apoptosis through induction of endoplasmic reticulum stress and Ca^2+^-dependent mitochondrial death pathway in human nasopharyngeal carcinoma cells. Anticancer Res..

[50] Chen Y.Y., Chiang S.Y., Lin J.G., Ma Y.S., Liao C.L., Weng S.W., Lai T.Y., Chung J.G. (2010). Emodin, aloe-emodin and rhein inhibit migration and invasion in human tongue cancer SCC-4 cells through the inhibition of gene expression of matrix metalloproteinase-9. Int. J. Oncol..

[51] Chen Y.Y., Chiang S.Y., Lin J.G., Yang J.S., Ma Y.S., Liao C.L., Lai T.Y., Tang N.Y., Chung J.G. (2010). Emodin, aloe-emodin and rhein induced DNA damage and inhibited DNA repair gene expression in SCC-4 human tongue cancer cells. Anticancer Res..

[52] Yang L., Lin S., Kang Y., Xiang Y., Xu L., Li J., Dai X., Liang G., Huang X., Zhao C. (2019). Rhein sensitizes human pancreatic cancer cells to EGFR inhibitors by inhibiting STAT3 pathway. J. Exp. Clin. Cancer Res..

[53] Zhou G., Peng F., Zhong Y., Chen Y., Tang M., Li D. (2017). Rhein suppresses matrix metalloproteinase production by regulating the Rac1/ROS/MAPK/AP-1 pathway in human ovarian carcinoma cells. Int. J. Oncol..

[54] Shi P., Huang Z., Chen G. (2008). Rhein induces apoptosis and cell cycle arrest in human hepatocellular carcinoma BEL-7402 cells. Am. J. Chin. Med..

[55] Wang A., Jiang H., Liu Y., Chen J., Zhou X., Zhao C., Chen X., Lin M. (2020). Rhein induces liver cancer cells apoptosis via activating ROS-dependent JNK/Jun/caspase-3 signaling pathway. J. Cancer.

[56] He Z.H., He M.F., Ma S.C., But P.P. (2009). Anti-angiogenic effects of rhubarb and its anthraquinone derivatives. J. Ethnopharmacol..

[57] Chai S., To K.K., Lin G. (2010). Circumvention of multi-drug resistance of cancer cells by Chinese herbal medicines. Chin. Med..

[58] Xu H., Lu Y., Zhang T., Liu K., Liu L., He Z., Xu B., Wu X. (2019). Characterization of binding interactions of anthraquinones and bovine beta-lactoglobulin. Food Chem..

[59] Feng H., Zhu Y., Fu Z., Li D. (2017). Preparation, characterization, and in vivo study of rhein solid lipid nanoparticles for oral delivery. Chem. Biol. Drug Des..

[60] Gómez-Gaete C., Retamal M., Chávez C., Bustos P., Godoy R., Torres-Vergara P. (2017). Development, characterization and in vitro evaluation of biodegradable rhein-loaded microparticles for treatment of osteoarthritis. Eur. J. Pharm. Sci..

[61] Yuan Z., Gu X. (2015). Preparation, characterization, and in vivo study of rhein-loaded poly (lactic-co-glycolic acid) nanoparticles for oral delivery. Drug Des. Dev..

[62] Kunnumakkara A.B., Sailo B.L., Banik K., Harsha C., Prasad S., Gupta S.C., Bharti A.C., Aggarwal B.B. (2018). Chronic diseases, inflammation, and spices: How are they linked?. J. Transl. Med..

[63] Khwairakpam A.D., Damayenti Y.D., Deka A., Monisha J., Roy N.K., Padmavathi G., Kunnumakkara A.B. (2018). Acorus calamus: A bio-reserve of medicinal values. J. Basic Clin. Physiol. Pharmacol..

[64] Kunnumakkara A.B., Sung B., Ravindran J., Diagaradjane P., Deorukhkar A., Dey S., Koca C., Tong Z., Gelovani J.G., Guha S. (2012). Zyflamend suppresses growth and sensitizes human pancreatic tumors to gemcitabine in an orthotopic mouse model through modulation of multiple targets. Int. J. Cancer.

[65] Rokaya M.B., Munzbergova Z., Timsina B., Bhattarai K.R. (2012). *Rheum* australe D. Don: A review of its botany, ethnobotany, phytochemistry and pharmacology. J. Ethnopharmacol..

[66] Yang F., Zhang T., Xu G., Chou F.E., Ito Y. (2001). pH-MODULATED STEPWISE ELUTION CCC AND ITS APPLICATION TO THE PREPARATIVE SEPARATION OF HYDROXYANTHRAQUINONE COMPOUNDS FROM TRADITIONAL CHINESE MEDICINAL HERBS. J. Liq. Chromatogr. Relat. Technol..

[67] Yang D.Y., Fushimi H., Cai S.Q., Komatsu K. (2004). Molecular analysis of *Rheum* species used as Rhei Rhizoma based on the chloroplast matK gene sequence and its application for identification. Biol. Pharm. Bull..

[68] Cojocaru A., Vlase L., Munteanu N., Stan T., Teliban G.C., Burducea M., Stoleru V. (2020). Dynamic of phenolic compounds, antioxidant activity, and yield of rhubarb under chemical, organic and biological fertilization. Plants.

[69] Duraipandiyan V., Baskar A.A., Ignacimuthu S., Muthukumar C., Al-Harbi N.A. (2012). Anticancer activity of Rhein isolated from Cassia fistula L. flower. Asian Pac. J. Trop. Dis..

[70] Duraipandiyan V., Ignacimuthu S., Gabriel Paulraj M. (2011). Antifeedant and larvicidal activities of Rhein isolated from the flowers of *Cassia fistula* L.. Saudi J. Biol. Sci..

[71] Yang F., Xu Y., Xiong A., He Y., Yang L., Wan Y.J., Wang Z. (2012). Evaluation of the protective effect of Rhei Radix et Rhizoma against alpha-naphthylisothiocyanate induced liver injury based on metabolic profile of bile acids. J. Ethnopharmacol..

[72] Harsha C., Banik K., Bordoloi D., Kunnumakkara A.B. (2017). Antiulcer properties of fruits and vegetables: A mechanism based perspective. Food Chem. Toxicol. Int. J. Publ. Br. Ind. Biol. Res. Assoc..

[73] Chinese Pharmacopoeia Commission (1997). Pharmacopoeia of the People’s Republic of China.

[74] Choi R.Y., Lee H.I., Ham J.R., Yee S.T., Kang K.Y., Lee M.K. (2018). Heshouwu (*Polygonum multiflorum* Thunb.) ethanol extract suppresses pre-adipocytes differentiation in 3T3-L1 cells and adiposity in obese mice. Biomed. Pharmacother..

[75] Lin L., Ni B., Lin H., Zhang M., Li X., Yin X., Qu C., Ni J. (2015). Traditional usages, botany, phytochemistry, pharmacology and toxicology of *Polygonum multiflorum* Thunb.: A review. J. Ethnopharmacol..

[76] Zhou H., Jiao D. (1990). 312 cases of gastric and duodenal ulcer bleeding treated with 3 kinds of alcoholic extract rhubarb tablets. Zhong Xi Yi Jie He Za Zhi = Chin. J. Mod. Dev. Tradit. Med..

[77] Wu J., Hu Y., Xiang L., Li S., Yuan Y., Chen X., Zhang Y., Huang W., Meng X., Wang P. (2016). San-Huang-Xie-Xin-Tang constituents exert drug-drug interaction of mutual reinforcement at both pharmacodynamics and pharmacokinetic level: A review. Front. Pharmacol..

[78] Ma B.L., Ma Y.M., Yan D.M., Zhou H., Shi R., Wang T.M., Yang Y., Wang C.H., Zhang N. (2009). Effective constituents in Xiexin Decoction for anti-inflammation. J. Ethnopharmacol..

[79] Yan S., Yue Y., Wang J., Li W., Sun M., Zeng L., Wang X. (2019). Banxia Xiexin decoction, a traditional Chinese medicine, alleviates colon cancer in nude mice. Ann. Transl. Med..

[80] Kim H.R., Lee G.S., Kim M.S., Ryu D.G., So H.S., Moon H.C., Lee Y.R., Yang S.H., Kwon K.B. (2018). Effects of Banxia Xiexin Decoction (半夏泻心汤) on Cisplatin-Induced Apoptosis of Human A549 Lung Cancer Cells. Chin. J. Integr. Med..

[81] Zhang A., Sun H., Yuan Y., Sun W., Jiao G., Wang X. (2011). An in vivo analysis of the therapeutic and synergistic properties of Chinese medicinal formula Yin-Chen-Hao-Tang based on its active constituents. Fitoterapia.

[82] Ibrahim M., Khan A.A., Tiwari S.K., Habeeb M.A., Khaja M.N., Habibullah C.M. (2006). Antimicrobial activity of Sapindus mukorossi and *Rheum* emodi extracts against H pylori: In vitro and in vivo studies. World J. Gastroenterol..

[83] Xu W., Hu M., Zhang Q., Yu J., Su W. (2018). Effects of anthraquinones from *Cassia occidentalis* L. on ovalbumin-induced airways inflammation in a mouse model of allergic asthma. J. Ethnopharmacol..

[84] Wei Y., Zhang T., Ito Y. (2003). Preparative separation of rhein from Chinese traditional herb by repeated high-speed counter-current chromatography. J. Chromatogr. A.

[85] Petralito S., Zanardi I., Memoli A., Annesini M.C., Travagli V. (2009). Solubility, spectroscopic properties and photostability of Rhein/cyclodextrin inclusion complex. Spectrochim. Acta. Part Amol. Biomol. Spectrosc..

[86] Jin Q., Jiang C., Gao M., Zhang D., Yao N., Feng Y., Wu T., Zhang J. (2019). Target exploration of rhein as a small-molecule necrosis avid agent by post-treatment click modification. New J. Chem..

[87] Zhang A., Wu T., Bian L., Li P., Liu Q., Zhang D., Jin Q., Zhang J., Huang G., Song S. (2019). Synthesis and Evaluation of Ga-68-Labeled Rhein for Early Assessment of Treatment-Induced Tumor Necrosis. Mol. Imaging Biol..

[88] Hwang D.S., Gu P.S., Kim N., Jang Y.P., Oh M.S. (2018). Effects of Rhei Undulati Rhizoma on lipopolysaccharide-induced neuroinflammation in vitro and *in vivo*. Environ. Toxicol..

[89] Liu Z., Lang Y., Li L., Liang Z., Deng Y., Fang R., Meng Q. (2018). Effect of emodin on chondrocyte viability in an in vitro model of osteoarthritis. Exp. Ther. Med..

[90] Qin F., Huang J., Huang X., Ren P. (2011). Simultaneous determination and pharmacokinetic comparisons of aloe-emodin, rhein, emodin, and chrysophanol after oral administration of these monomers, rhei rhizoma and chaiqin-chengqi-tang, to rats. J. Liq. Chromatogr. Relat. Technol..

[91] Zhao Q., Wang X., Chen A., Cheng X., Zhang G., Sun J., Zhao Y., Huang Y., Zhu Y. (2018). Rhein protects against cerebral ischemic-/reperfusion-induced oxidative stress and apoptosis in rats. Int. J. Mol. Med..

[92] Zhang Q., Liu L., Lin W., Yin S., Duan A., Liu Z., Cao W. (2017). Rhein reverses Klotho repression via promoter demethylation and protects against kidney and bone injuries in mice with chronic kidney disease. Kidney Int..

[93] Cyong J., Matsumoto T., Arakawa K., Kiyohara H., Yamada H., Otsuka Y. (1987). Anti-Bacteroides fragilis substance from rhubarb. J. Ethnopharmacol..

[94] Vargas F., Díaz Y., Carbonell K. (2004). Antioxidant and Scavenging Activity of Emodin, Aloe-Emodin, and Rhein on Free-Radical and Reactive Oxygen Species. Pharm. Biol..

[95] Mendes A.F., Caramona M.M., De Carvalho A.P., Lopes M.C. (2002). Diacerhein and rhein prevent interleukin-1beta-induced nuclear factor-kappaB activation by inhibiting the degradation of inhibitor kappaB-alpha. Pharmacol. Toxicol..

[96] Kuo P.L., Hsu Y.L., Ng L.T., Lin C.C. (2004). Rhein inhibits the growth and induces the apoptosis of Hep G2 cells. Planta Med..

[97] You L., Dong X., Yin X., Yang C., Leng X., Wang W., Ni J. (2018). Rhein Induces Cell Death in HepaRG Cells through Cell Cycle Arrest and Apoptotic Pathway. Int. J. Mol. Sci..

[98] Yuan X., Tian W., Hua Y., Hu L., Yang J., Xie J., Hu J., Wang F. (2018). Rhein enhances the cytotoxicity of effector lymphocytes in colon cancer under hypoxic conditions. Exp. Ther. Med..

[99] Pearson G., Robinson F., Beers Gibson T., Xu B.E., Karandikar M., Berman K., Cobb M.H. (2001). Mitogen-activated protein (MAP) kinase pathways: Regulation and physiological functions. Endocr. Rev..

[100] Morrison D.K. (2012). MAP kinase pathways. Cold Spring Harb. Perspect. Biol..

[101] Dai X., Wang L., Deivasigamni A., Looi C.Y., Karthikeyan C., Trivedi P., Chinnathambi A., Alharbi S.A., Arfuso F., Dharmarajan A. (2017). A novel benzimidazole derivative, MBIC inhibits tumor growth and promotes apoptosis via activation of ROS-dependent JNK signaling pathway in hepatocellular carcinoma. Oncotarget.

[102] Kim S.M., Kim C., Bae H., Lee J.H., Baek S.H., Nam D., Chung W.S., Shim B.S., Lee S.G., Kim S.H. (2015). 6-Shogaol exerts anti-proliferative and pro-apoptotic effects through the modulation of STAT3 and MAPKs signaling pathways. Mol. Carcinog..

[103] Kannaiyan R., Manu K.A., Chen L., Li F., Rajendran P., Subramaniam A., Lam P., Kumar A.P., Sethi G. (2011). Celastrol inhibits tumor cell proliferation and promotes apoptosis through the activation of c-Jun N-terminal kinase and suppression of PI3 K/Akt signaling pathways. Apoptosis Int. J. Program. Cell Death.

[104] Woo C.C., Hsu A., Kumar A.P., Sethi G., Tan K.H. (2013). Thymoquinone inhibits tumor growth and induces apoptosis in a breast cancer xenograft mouse model: The role of p38 MAPK and ROS. PLoS ONE.

[105] Fu M., Wang C., Li Z., Sakamaki T., Pestell R.G. (2004). Minireview: Cyclin D1: Normal and abnormal functions. Endocrinology.

[106] Bhuvanalakshmi G., Gamit N., Patil M., Arfuso F., Sethi G., Dharmarajan A., Kumar A.P., Warrier S. (2018). Stemness, Pluripotentiality, and Wnt Antagonism: sFRP4, a Wnt antagonist Mediates Pluripotency and Stemness in Glioblastoma. Cancers.

[107] Bhuvanalakshmi G., Basappa, Rangappa K.S., Dharmarajan A., Sethi G., Kumar A.P., Warrier S. (2017). Breast Cancer Stem-Like Cells Are Inhibited by Diosgenin, a Steroidal Saponin, by the Attenuation of the Wnt beta-Catenin Signaling via the Wnt Antagonist Secreted Frizzled Related Protein-4. Front. Pharmacol..

[108] Panda P.K., Naik P.P., Praharaj P.P., Meher B.R., Gupta P.K., Verma R.S., Maiti T.K., Shanmugam M.K., Chinnathambi A., Alharbi S.A. (2018). Abrus agglutinin stimulates BMP-2-dependent differentiation through autophagic degradation of beta-catenin in colon cancer stem cells. Mol. Carcinog..

[109] Fang D., Hawke D., Zheng Y., Xia Y., Meisenhelder J., Nika H., Mills G.B., Kobayashi R., Hunter T., Lu Z. (2007). Phosphorylation of beta-catenin by AKT promotes beta-catenin transcriptional activity. J. Biol. Chem..

[110] Hsia T.C., Yang J.S., Chen G.W., Chiu T.H., Lu H.F., Yang M.D., Yu F.S., Liu K.C., Lai K.C., Lin C.C. (2009). The roles of endoplasmic reticulum stress and Ca^2+^ on rhein-induced apoptosis in A-549 human lung cancer cells. Anticancer Res..

[111] Liu S., Wang J., Shao T., Song P., Kong Q., Hua H., Luo T., Jiang Y. (2018). The natural agent rhein induces beta-catenin degradation and tumour growth arrest. J. Cell. Mol. Med..

[112] Puar Y.R., Shanmugam M.K., Fan L., Arfuso F., Sethi G., Tergaonkar V. (2018). Evidence for the Involvement of the Master Transcription Factor NF-kappaB in Cancer Initiation and Progression. Biomedicines.

[113] Shin E.M., Hay H.S., Lee M.H., Goh J.N., Tan T.Z., Sen Y.P., Lim S.W., Yousef E.M., Ong H.T., Thike A.A. (2014). DEAD-box helicase DP103 defines metastatic potential of human breast cancers. J. Clin. Investig..

[114] Ahn K.S., Sethi G., Chaturvedi M.M., Aggarwal B.B. (2008). Simvastatin, 3-hydroxy-3-methylglutaryl coenzyme A reductase inhibitor, suppresses osteoclastogenesis induced by receptor activator of nuclear factor-kappaB ligand through modulation of NF-kappaB pathway. Int. J. Cancer.

[115] Sethi G., Ahn K.S., Sung B., Aggarwal B.B. (2008). Pinitol targets nuclear factor-kappaB activation pathway leading to inhibition of gene products associated with proliferation, apoptosis, invasion, and angiogenesis. Mol. Cancer Ther..

[116] Sawhney M., Rohatgi N., Kaur J., Shishodia S., Sethi G., Gupta S.D., Deo S.V., Shukla N.K., Aggarwal B.B., Ralhan R. (2007). Expression of NF-kappaB parallels COX-2 expression in oral precancer and cancer: Association with smokeless tobacco. Int. J. Cancer.

[117] Ahn K.S., Sethi G., Jain A.K., Jaiswal A.K., Aggarwal B.B. (2006). Genetic deletion of NAD(P)H:quinone oxidoreductase 1 abrogates activation of nuclear factor-kappaB, IkappaBalpha kinase, c-Jun N-terminal kinase, Akt, p38, and p44/42 mitogen-activated protein kinases and potentiates apoptosis. J. Biol. Chem..

[118] Monisha J., Roy N.K., Bordoloi D., Kumar A., Golla R., Kotoky J., Padmavathi G., Kunnumakkara A.B. (2017). Nuclear Factor Kappa B: A Potential Target to Persecute Head and Neck Cancer. Curr. Drug Targets.

[119] Ahn K.S., Sethi G., Aggarwal B.B. (2008). Reversal of chemoresistance and enhancement of apoptosis by statins through down-regulation of the NF-kappaB pathway. Biochem. Pharmacol..

[120] Manna S.K., Aggarwal R.S., Sethi G., Aggarwal B.B., Ramesh G.T. (2007). Morin (3,5,7,2’,4’-Pentahydroxyflavone) abolishes nuclear factor-kappaB activation induced by various carcinogens and inflammatory stimuli, leading to suppression of nuclear factor-kappaB-regulated gene expression and up-regulation of apoptosis. Clin. Cancer Res. Off. J. Am. Assoc. Cancer Res..

[121] Chua A.W., Hay H.S., Rajendran P., Shanmugam M.K., Li F., Bist P., Koay E.S., Lim L.H., Kumar A.P., Sethi G. (2010). Butein downregulates chemokine receptor CXCR4 expression and function through suppression of NF-kappaB activation in breast and pancreatic tumor cells. Biochem. Pharmacol..

[122] Shanmugam M.K., Manu K.A., Ong T.H., Ramachandran L., Surana R., Bist P., Lim L.H., Kumar A.P., Hui K.M., Sethi G. (2011). Inhibition of CXCR4/CXCL12 signaling axis by ursolic acid leads to suppression of metastasis in transgenic adenocarcinoma of mouse prostate model. Int. J. Cancer.

[123] Siveen K.S., Mustafa N., Li F., Kannaiyan R., Ahn K.S., Kumar A.P., Chng W.J., Sethi G. (2014). Thymoquinone overcomes chemoresistance and enhances the anticancer effects of bortezomib through abrogation of NF-kappaB regulated gene products in multiple myeloma xenograft mouse model. Oncotarget.

[124] Kunnumakkara A.B., Guha S., Krishnan S., Diagaradjane P., Gelovani J., Aggarwal B.B. (2007). Curcumin potentiates antitumor activity of gemcitabine in an orthotopic model of pancreatic cancer through suppression of proliferation, angiogenesis, and inhibition of nuclear factor-κB–regulated gene products. Cancer Res..

[125] Kunnumakkara A.B., Nair A.S., Ahn K.S., Pandey M.K., Yi Z., Liu M., Aggarwal B.B. (2007). Gossypin, a pentahydroxy glucosyl flavone, inhibits the transforming growth factor beta-activated kinase-1-mediated NF-kappaB activation pathway, leading to potentiation of apoptosis, suppression of invasion, and abrogation of osteoclastogenesis. Blood.

[126] Li F., Shanmugam M.K., Siveen K.S., Wang F., Ong T.H., Loo S.Y., Swamy M.M., Mandal S., Kumar A.P., Goh B.C. (2015). Garcinol sensitizes human head and neck carcinoma to cisplatin in a xenograft mouse model despite downregulation of proliferative biomarkers. Oncotarget.

[127] Li F., Shanmugam M.K., Chen L., Chatterjee S., Basha J., Kumar A.P., Kundu T.K., Sethi G. (2013). Garcinol, a polyisoprenylated benzophenone modulates multiple proinflammatory signaling cascades leading to the suppression of growth and survival of head and neck carcinoma. Cancer Prev. Res..

[128] Manu K.A., Shanmugam M.K., Ramachandran L., Li F., Fong C.W., Kumar A.P., Tan P., Sethi G. (2012). First evidence that gamma-tocotrienol inhibits the growth of human gastric cancer and chemosensitizes it to capecitabine in a xenograft mouse model through the modulation of NF-kappaB pathway. Clin. Cancer Res. Off. J. Am. Assoc. Cancer Res..

[129] Manu K.A., Shanmugam M.K., Li F., Chen L., Siveen K.S., Ahn K.S., Kumar A.P., Sethi G. (2014). Simvastatin sensitizes human gastric cancer xenograft in nude mice to capecitabine by suppressing nuclear factor-kappa B-regulated gene products. J. Mol. Med..

[130] Manu K.A., Shanmugam M.K., Ramachandran L., Li F., Siveen K.S., Chinnathambi A., Zayed M.E., Alharbi S.A., Arfuso F., Kumar A.P. (2015). Isorhamnetin augments the *anti*-tumor effect of capecitabine through the negative regulation of NF-kappaB signaling cascade in gastric cancer. Cancer Lett..

[131] Ghosh S., May M.J., Kopp E.B. (1998). NF-kappa B and Rel proteins: Evolutionarily conserved mediators of immune responses. Annu. Rev. Immunol..

[132] Kunnumakkara A.B., Diagaradjane P., Guha S., Deorukhkar A., Shentu S., Aggarwal B.B., Krishnan S. (2008). Curcumin sensitizes human colorectal Cancer xenografts in nude mice to γ-radiation by targeting nuclear factor-κB–regulated gene products. Clin. Cancer Res..

[133] Lin Y.G., Kunnumakkara A.B., Nair A., Merritt W.M., Han L.Y., Armaiz-Pena G.N., Kamat A.A., Spannuth W.A., Gershenson D.M., Lutgendorf S.K. (2007). Curcumin inhibits tumor growth and angiogenesis in ovarian carcinoma by targeting the nuclear factor-κB pathway. Clin. Cancer Res..

[134] Heymach J.V., Shackleford T.J., Tran H.T., Yoo S.Y., Do K.A., Wergin M., Saintigny P., Vollmer R.T., Polascik T.J., Snyder D.C. (2011). Effect of low-fat diets on plasma levels of NF-kappaB-regulated inflammatory cytokines and angiogenic factors in men with prostate cancer. Cancer Prev. Res..

[135] Gao Y., Chen X., Fang L., Liu F., Cai R., Peng C., Qi Y. (2014). Rhein exerts pro- and anti-inflammatory actions by targeting IKKbeta inhibition in LPS-activated macrophages. Free Radic. Biol. Med..

[136] Zhao X.Y., Chen T.T., Xia L., Guo M., Xu Y., Yue F., Jiang Y., Chen G.Q., Zhao K.W. (2010). Hypoxia inducible factor-1 mediates expression of galectin-1: The potential role in migration/invasion of colorectal cancer cells. Carcinogenesis.

[137] Clambey E.T., McNamee E.N., Westrich J.A., Glover L.E., Campbell E.L., Jedlicka P., De Zoeten E.F., Cambier J.C., Stenmark K.R., Colgan S.P. (2012). Hypoxia-inducible factor-1 alpha-dependent induction of FoxP3 drives regulatory T-cell abundance and function during inflammatory hypoxia of the mucosa. Proc. Natl. Acad. Sci. USA.

[138] Fernand V.E., Losso J.N., Truax R.E., Villar E.E., Bwambok D.K., Fakayode S.O., Lowry M., Warner I.M. (2011). Rhein inhibits angiogenesis and the viability of hormone-dependent and -independent cancer cells under normoxic or hypoxic conditions in vitro. Chem. Biol. Interact..

[139] Kunnumakkara A.B., Diagaradjane P., Anand P., Kuzhuvelil H.B., Deorukhkar A., Gelovani J., Guha S., Krishnan S., Aggarwal B.B. (2009). Curcumin sensitizes human colorectal cancer to capecitabine by modulation of cyclin D1, COX-2, MMP-9, VEGF and CXCR4 expression in an orthotopic mouse model. Int. J. Cancer.

[140] Roy N.K., Bordoloi D., Monisha J., Padmavathi G., Kotoky J., Golla R., Kunnumakkara A.B. (2017). Specific Targeting of Akt Kinase Isoforms: Taking the Precise Path for Prevention and Treatment of Cancer. Curr. Drug Targets.

[141] Bao L., Kimzey A., Sauter G., Sowadski J.M., Lu K.P., Wang D.G. (2004). Prevalent overexpression of prolyl isomerase Pin1 in human cancers. Am. J. Pathol..

[142] Cho J.H., Chae J.I., Shim J.H. (2017). Rhein exhibits antitumorigenic effects by interfering with the interaction between prolyl isomerase Pin1 and c-Jun. Oncol. Rep..

[143] Banik K., Ranaware A.M., Deshpande V., Nalawade S.P., Padmavathi G., Bordoloi D., Sailo B.L., Shanmugam M.K., Fan L., Arfuso F. (2019). Honokiol for cancer therapeutics: A traditional medicine that can modulate multiple oncogenic targets. Pharmacol. Res..

[144] Wang J., Liu S., Yin Y., Li M., Wang B., Yang L., Jiang Y. (2015). FOXO3-mediated up-regulation of Bim contributes to rhein-induced cancer cell apoptosis. Apoptosis Int. J. Program. Cell Death.

[145] Lin S., Fujii M., Hou D.X. (2003). Rhein induces apoptosis in HL-60 cells via reactive oxygen species-independent mitochondrial death pathway. Arch. Biochem. Biophys..

[146] Shen Z., Zhu B., Li J., Qin L. (2019). Rhein Augments Antiproliferative Effects of Atezolizumab Based on Breast Cancer (4T1) Regression. Planta Med..

[147] Lin Y.J., Huang Y.H., Zhen Y.Z., Liu X.J., Zhen Y.S. (2008). Rhein lysinate induces apoptosis in breast cancer SK-Br-3 cells by inhibiting HER-2 signal pathway. Yao Xue Xue Bao = Acta Pharm. Sin..

[148] Liu Y., Zhong Y., Tian W., Lan F., Kang J., Pang H., Hou H., Li D. (2019). An autophagy-dependent cell death of MDA-MB-231 cells triggered by a novel Rhein derivative 4F. Anti-Cancer Drugs.

[149] Ip S.W., Weng Y.S., Lin S.Y., Mei D., Tang N.Y., Su C.C., Chung J.G. (2007). The role of Ca^+2^ on rhein-induced apoptosis in human cervical cancer Ca Ski cells. Anticancer. Res..

[150] Aviello G., Rowland I., Gill C.I., Acquaviva A.M., Capasso F., McCann M., Capasso R., Izzo A.A., Borrelli F. (2010). Anti-proliferative effect of rhein, an anthraquinone isolated from Cassia species, on Caco-2 human adenocarcinoma cells. J. Cell. Mol. Med..

[151] Zhuang Y., Bai Y., Hu Y., Guo Y., Xu L., Hu W., Yang L., Zhao C., Li X., Zhao H. (2019). Rhein sensitizes human colorectal cancer cells to EGFR inhibitors by inhibiting STAT3 pathway. Oncotargets Ther..

[152] Tang N., Chang J., Lu H.C., Zhuang Z., Cheng H.L., Shi J.X., Rao J. (2017). Rhein induces apoptosis and autophagy in human and rat glioma cells and mediates cell differentiation by ERK inhibition. Microb. Pathog..

[153] Chen J., Luo B., Wen S., Pi R. (2019). Discovery of a novel rhein-SAHA hybrid as a multi-targeted anti-glioblastoma drug. Investig. New Drugs.

[154] Gu L., Zhang H., Liu T., Draganov A., Yi S., Wang B., Zhou M. (2018). Inhibition of MDM2 by a Rhein-Derived Compound AQ-101 Suppresses Cancer Development in SCID Mice. Mol. Cancer Ther..

[155] Heo S.K., Noh E.K., Kim J.Y., Jegal S., Jeong Y., Cheon J., Koh S., Baek J.H., Min Y.J., Choi Y. (2018). Rhein augments ATRA-induced differentiation of acute promyelocytic leukemia cells. Phytomed. Int. J. Phytother. Phytopharm..

[156] Wu L., Cao K., Ni Z., Wang S., Li W., Liu X., Chen Z. (2019). Rhein reverses doxorubicin resistance in SMMC-7721 liver cancer cells by inhibiting energy metabolism and inducing mitochondrial permeability transition pore opening. Biofactors.

[157] Yang L., Li J., Xu L., Lin S., Xiang Y., Dai X., Liang G., Huang X., Zhu J., Zhao C. (2019). Rhein shows potent efficacy against non-small-cell lung cancer through inhibiting the STAT3 pathway. Cancer Manag. Res..

[158] Bu T., Wang C., Jin H., Meng Q., Huo X., Sun H., Sun P., Wu J., Ma X., Liu Z. (2020). Organic anion transporters and PI3K-AKT-mTOR pathway mediate the synergistic anticancer effect of pemetrexed and rhein. J. Cell. Physiol..

[159] Ren B., Guo W., Tang Y., Zhang J., Xiao N., Zhang L., Li W. (2019). Rhein Inhibits the Migration of Ovarian Cancer Cells through Down-Regulation of Matrix Metalloproteinases. Biol. Pharm. Bull..

[160] Hu L., Cui R., Liu H., Wang F. (2017). Emodin and rhein decrease levels of hypoxia-inducible factor-1alpha in human pancreatic cancer cells and attenuate cancer cachexia in athymic mice carrying these cells. Oncotarget.

[161] Shanmugam M.K., Ahn K.S., Hsu A., Woo C.C., Yuan Y., Tan K.H.B., Chinnathambi A., Alahmadi T.A., Alharbi S.A., Koh A.P.F. (2018). Thymoquinone Inhibits Bone Metastasis of Breast Cancer Cells Through Abrogation of the CXCR4 Signaling Axis. Front. Pharmacol..

[162] Liu L., Ahn K.S., Shanmugam M.K., Wang H., Shen H., Arfuso F., Chinnathambi A., Alharbi S.A., Chang Y., Sethi G. (2019). Oleuropein induces apoptosis via abrogating NF-kappaB activation cascade in estrogen receptor-negative breast cancer cells. J. Cell. Biochem..

[163] Wang C., Kar S., Lai X., Cai W., Arfuso F., Sethi G., Lobie P.E., Goh B.C., Lim L.H.K., Hartman M. (2018). Triple negative breast cancer in Asia: An insider’s view. Cancer Treat. Rev..

[164] Mohan C.D., Srinivasa V., Rangappa S., Mervin L., Mohan S., Paricharak S., Baday S., Li F., Shanmugam M.K., Chinnathambi A. (2016). Trisubstituted-Imidazoles Induce Apoptosis in Human Breast Cancer Cells by Targeting the Oncogenic PI3K/Akt/mTOR Signaling Pathway. PLoS ONE.

[165] Jia L.Y., Shanmugam M.K., Sethi G., Bishayee A. (2016). Potential role of targeted therapies in the treatment of triple-negative breast cancer. Anti-Cancer Drugs.

[166] Ningegowda R., Shivananju N.S., Rajendran P., Basappa, Rangappa K.S., Chinnathambi A., Li F., Achar R.R., Shanmugam M.K., Bist P. (2017). A novel 4,6-disubstituted-1,2,4-triazolo-1,3,4-thiadiazole derivative inhibits tumor cell invasion and potentiates the apoptotic effect of TNFalpha by abrogating NF-kappaB activation cascade. Apoptosis Int. J. Program. Cell Death.

[167] Bhojwani D., Yang J.J., Pui C.H. (2015). Biology of childhood acute lymphoblastic leukemia. Pediatric Clin. N. Am..

[168] Stewart B.W., Wild C. (2014). World Cancer Report 2014.

[169] Sethi G., Chatterjee S., Rajendran P., Li F., Shanmugam M.K., Wong K.F., Kumar A.P., Senapati P., Behera A.K., Hui K.M. (2014). Inhibition of STAT3 dimerization and acetylation by garcinol suppresses the growth of human hepatocellular carcinoma in vitro and in vivo. Mol. Cancer.

[170] Siveen K.S., Ahn K.S., Ong T.H., Shanmugam M.K., Li F., Yap W.N., Kumar A.P., Fong C.W., Tergaonkar V., Hui K.M. (2014). Y-tocotrienol inhibits angiogenesis-dependent growth of human hepatocellular carcinoma through abrogation of AKT/mTOR pathway in an orthotopic mouse model. Oncotarget.

[171] Mohan C.D., Bharathkumar H., Bulusu K.C., Pandey V., Rangappa S., Fuchs J.E., Shanmugam M.K., Dai X., Li F., Deivasigamani A. (2014). Development of a novel azaspirane that targets the Janus kinase-signal transducer and activator of transcription (STAT) pathway in hepatocellular carcinoma in vitro and in vivo. J. Biol. Chem..

[172] Dai X., Ahn K.S., Kim C., Siveen K.S., Ong T.H., Shanmugam M.K., Li F., Shi J., Kumar A.P., Wang L.Z. (2015). Ascochlorin, an isoprenoid antibiotic inhibits growth and invasion of hepatocellular carcinoma by targeting STAT3 signaling cascade through the induction of PIAS3. Mol. Oncol..

[173] Rajendran P., Li F., Shanmugam M.K., Vali S., Abbasi T., Kapoor S., Ahn K.S., Kumar A.P., Sethi G. (2012). Honokiol inhibits signal transducer and activator of transcription-3 signaling, proliferation, and survival of hepatocellular carcinoma cells via the protein tyrosine phosphatase SHP-1. J. Cell. Physiol..

[174] Rajendran P., Li F., Manu K.A., Shanmugam M.K., Loo S.Y., Kumar A.P., Sethi G. (2011). gamma-Tocotrienol is a novel inhibitor of constitutive and inducible STAT3 signalling pathway in human hepatocellular carcinoma: Potential role as an antiproliferative, pro-apoptotic and chemosensitizing agent. Br. J. Pharmacol..

[175] Tan S.M., Li F., Rajendran P., Kumar A.P., Hui K.M., Sethi G. (2010). Identification of beta-escin as a novel inhibitor of signal transducer and activator of transcription 3/Janus-activated kinase 2 signaling pathway that suppresses proliferation and induces apoptosis in human hepatocellular carcinoma cells. J. Pharmacol. Exp. Ther..

[176] Singh A.K., Roy N.K., Anip A., Banik K., Monisha J., Bordoloi D., Kunnumakkara A.B. (2018). Different methods to inhibit chemoresistance in Hepatocellular carcinoma. Cancer Cell Chemoresistance and Chemosensitization.

[177] Wang L., Syn N.L., Subhash V.V., Any Y., Thuya W.L., Cheow E.S.H., Kong L., Yu F., Peethala P.C., Wong A.L. (2018). Pan-HDAC inhibition by panobinostat mediates chemosensitization to carboplatin in non-small cell lung cancer via attenuation of EGFR signaling. Cancer Lett..

[178] Jung Y.Y., Shanmugam M.K., Narula A.S., Kim C., Lee J.H., Namjoshi O.A., Blough B.E., Sethi G., Ahn K.S. (2019). Oxymatrine Attenuates Tumor Growth and Deactivates STAT5 Signaling in a Lung Cancer Xenograft Model. Cancers.

[179] Lee J.H., Mohan C.D., Basappa S., Rangappa S., Chinnathambi A., Alahmadi T.A., Alharbi S.A., Kumar A.P., Sethi G., Ahn K.S. (2019). The IkappaB Kinase Inhibitor ACHP Targets the STAT3 Signaling Pathway in Human Non-Small Cell Lung Carcinoma Cells. Biomolecules.

[180] Lee J.H., Chinnathambi A., Alharbi S.A., Shair O.H.M., Sethi G., Ahn K.S. (2019). Farnesol abrogates epithelial to mesenchymal transition process through regulating Akt/mTOR pathway. Pharmacol. Res..

[181] Baek S.H., Ko J.H., Lee J.H., Kim C., Lee H., Nam D., Lee J., Lee S.G., Yang W.M., Um J.Y. (2017). Ginkgolic Acid Inhibits Invasion and Migration and TGF-beta-Induced EMT of Lung Cancer Cells Through PI3K/Akt/mTOR Inactivation. J. Cell. Physiol..

[182] Ong P.S., Wang L., Chia D.M., Seah J.Y., Kong L.R., Thuya W.L., Chinnathambi A., Lau J.Y., Wong A.L., Yong W.P. (2016). A novel combinatorial strategy using Seliciclib((R)) and Belinostat((R)) for eradication of non-small cell lung cancer via apoptosis induction and BID activation. Cancer Lett..

[183] Lee J.H., Kim C., Sethi G., Ahn K.S. (2015). Brassinin inhibits STAT3 signaling pathway through modulation of PIAS-3 and SOCS-3 expression and sensitizes human lung cancer xenograft in nude mice to paclitaxel. Oncotarget.

[184] Lee J.H., Kim C., Lee S.G., Sethi G., Ahn K.S. (2018). Ophiopogonin D, a Steroidal Glycoside Abrogates STAT3 Signaling Cascade and Exhibits Anti-Cancer Activity by Causing GSH/GSSG Imbalance in Lung Carcinoma. Cancers.

[185] Xianghong S., Yuwei S., Hong L., Wei S. (2010). Influence of main component of Heshouwu such as emodin, rhein and toluylene glycoside on hepatic cells and hepatoma carcinoma cell. Mod. J. Integr. Tradit. Chin. West. Med..

[186] Mao Y., Zhang M., Yang J., Sun H., Wang D., Zhang X., Yu F., Li J. (2017). The UCP2-related mitochondrial pathway participates in rhein-induced apoptosis in HK-2 cells. Toxicol. Res..

[187] Bounda G.A., Zhou W., Wang D.D., Yu F. (2015). Rhein elicits in vitro cytotoxicity in primary human liver HL-7702 cells by inducing apoptosis through mitochondria-mediated pathway. Evid. Based Complementary Altern. Med..

[188] Roy N.K., Parama D., Banik K., Bordoloi D., Devi A.K., Thakur K.K., Padmavathi G., Shakibaei M., Fan L., Sethi G. (2019). An Update on Pharmacological Potential of Boswellic Acids against Chronic Diseases. Int. J. Mol. Sci..

[189] Abotaleb M., Kubatka P., Caprnda M., Varghese E., Zolakova B., Zubor P., Opatrilova R., Kruzliak P., Stefanicka P., Büsselberg D. (2018). Chemotherapeutic agents for the treatment of metastatic breast cancer: An update. Biomed. Pharmacother..

[190] Kapinova A., Stefanicka P., Kubatka P., Zubor P., Uramova S., Kello M., Mojzis J., Blahutova D., Qaradakhi T., Zulli A. (2017). Are plant-based functional foods better choice against cancer than single phytochemicals? A critical review of current breast cancer research. Biomed. Pharmacother..

[191] Harikumar K.B., Kunnumakkara A.B., Sethi G., Diagaradjane P., Anand P., Pandey M.K., Gelovani J., Krishnan S., Guha S., Aggarwal B.B. (2010). Resveratrol, a multitargeted agent, can enhance antitumor activity of gemcitabine in vitro and in orthotopic mouse model of human pancreatic cancer. Int. J. Cancer.

[192] Sung B., Kunnumakkara A.B., Sethi G., Anand P., Guha S., Aggarwal B.B. (2009). Curcumin circumvents chemoresistance in vitro and potentiates the effect of thalidomide and bortezomib against human multiple myeloma in nude mice model. Mol. Cancer. Ther..

[193] Kunnumakkara A.B., Bordoloi D., Sailo B.L., Roy N.K., Thakur K.K., Banik K., Shakibaei M., Gupta S.C., Aggarwal B.B. (2019). Cancer drug development: The missing links. Exp. Biol. Med..

